# Genome-wide identification of genes regulating DNA methylation using genetic anchors for causal inference

**DOI:** 10.1186/s13059-020-02114-z

**Published:** 2020-08-28

**Authors:** Paul J. Hop, René Luijk, Lucia Daxinger, Maarten van Iterson, Koen F. Dekkers, Rick Jansen, BIOS Consortium, Bastiaan T. Heijmans, Peter A. C. ’t Hoen, Joyce van Meurs, Rick Jansen, Lude Franke, Dorret I. Boomsma, René Pool, Jenny van Dongen, Jouke J. Hottenga, Marleen M. J. van Greevenbroek, Coen D. A. Stehouwer, Carla J. H. van der Kallen, Casper G. Schalkwijk, Cisca Wijmenga, Sasha Zhernakova, Ettje F. Tigchelaar, P. Eline Slagboom, Marian Beekman, Joris Deelen, Diana van Heemst, Jan H. Veldink, Leonard H. van den Berg, Cornelia M. van Duijn, Aaron Isaacs, André G. Uitterlinden, P. Mila Jhamai, Michael Verbiest, H. Eka D. Suchiman, Marijn Verkerk, Ruud van der Breggen, Jeroen van Rooij, Nico Lakenberg, Hailiang Mei, Maarten van Iterson, Dasha V. Zhernakova, Peter van ’t Hof, Patrick Deelen, Peter A. C. ’t Hoen, Martijn Vermaat, René Luijk, Marc Jan Bonder, Freerk van Dijk, Wibowo Arindrarto, Szymon M. Kielbasa, Erik. W. van Zwet, Peter-Bram ’t Hoen, Joyce B. J. van Meurs, Peter A. C. ’t Hoen, M. Arfan Ikram, Marleen M. J. van Greevenbroek, Dorret I. Boomsma, P. Eline Slagboom, Jan H. Veldink, Erik W. van Zwet, Bastiaan T. Heijmans

**Affiliations:** 1 Molecular Epidemiology, Department of Biomedical Data Sciences, Leiden University Medical Center, 2333 ZC Leiden, The Netherlands; 2 Department of Neurology, UMC Utrecht Brain Center, University Medical Centre Utrecht, Utrecht University, 3584 CG Utrecht, The Netherlands; 3 Department of Human Genetics, Leiden University Medical Center, 2333 ZC Leiden, The Netherlands; 4 Department of Psychiatry, Amsterdam UMC, Vrije Universiteit Amsterdam, Amsterdam Neuroscience, 1081 HV Amsterdam, The Netherlands; 5 Department of Internal Medicine, Erasmus Medical Centre, Rotterdam, The Netherlands; 6 Centre for Molecular and Biomolecular Informatics, Radboud Institute for Molecular Life Sciences, Radboud University Medical Center Nijmegen, Nijmegen, The Netherlands; 7 Department of Epidemiology, Erasmus University Medical Center, 3015 CE Rotterdam, The Netherlands; 8 Department of Internal Medicine, Maastricht University Medical Center, 6211 LK Maastricht, The Netherlands; 9 School for Cardiovascular Diseases (CARIM), Maastricht University Medical Center, 6229 ER Maastricht, The Netherlands; 10 Department of Biological Psychology, Vrije Universiteit Amsterdam, Neuroscience Campus Amsterdam, 1081 BT Amsterdam, The Netherlands; 11 Medical Statistics, Department of Biomedical Data Sciences, Leiden University Medical Center, 2333 ZC Leiden, Zuid-Holland The Netherlands

**Keywords:** DNA methylation, Epigenetic regulation, Transcription factor, Chromatin, Genetic instrumental variable, Functional genomics, Pleiotropy, Causal inference

## Abstract

**Background:**

DNA methylation is a key epigenetic modification in human development and disease, yet there is limited understanding of its highly coordinated regulation. Here, we identify 818 genes that affect DNA methylation patterns in blood using large-scale population genomics data.

**Results:**

By employing genetic instruments as causal anchors, we establish directed associations between gene expression and distant DNA methylation levels, while ensuring specificity of the associations by correcting for linkage disequilibrium and pleiotropy among neighboring genes. The identified genes are enriched for transcription factors, of which many consistently increased or decreased DNA methylation levels at multiple CpG sites. In addition, we show that a substantial number of transcription factors affected DNA methylation at their experimentally determined binding sites. We also observe genes encoding proteins with heterogenous functions that have widespread effects on DNA methylation, e.g., *NFKBIE*, *CDCA7(L)*, and *NLRC5*, and for several examples, we suggest plausible mechanisms underlying their effect on DNA methylation.

**Conclusion:**

We report hundreds of genes that affect DNA methylation and provide key insights in the principles underlying epigenetic regulation.

## Background

The epigenome is fundamental to development and cell differentiation. Dysregulation of the epigenome is a hallmark of many diseases, ranging from rare developmental disorders to common complex diseases and aging [1–3]. The epigenome is highly dynamic and is extensively modified and remodeled in response to external and internal stimuli [4]. However, the networks underlying these highly coordinated epigenetic modifications remain to be fully elucidated. Hence, the systematic identification of genes that are involved in epigenetic regulation and the determination of their respective target sites will be a crucial step towards an in-depth understanding of epigenomic (dys)regulation.

DNA methylation is a key component of the epigenome that controls, stabilizes, and/or marks the transcriptional potential of a genomic region [5]. It involves the addition of a methyl group onto cytosines, mainly at CpG dinucleotides. Although considerable research has been devoted to studying the enzymes that *write*, *maintain*, and *erase* DNA methylation (i.e., DNMTs and TETs) [6], less is known about factors that are otherwise involved in the regulation of DNA methylation. These may include proteins and non-coding RNAs that regulate, interact with, or recruit the DNA methylation machinery [7]. Transcription factors, for example, do not only act indirectly by regulating the transcription of epigenetic genes, but have also been indicated to control the DNA methylation state of their target sites by recruiting or repelling DNMT or TET proteins [8, 9]. Experimental evidence for genes involved in the regulation of DNA methylation has been mainly obtained from in vitro experiments focusing on single genes or is based on animal models [8, 10–12]. A comprehensive genome-wide survey of genes affecting DNA methylation in humans is currently lacking.

We recently developed a method to identify directed and specific gene-gene interactions in population omics data [13]. Instead of using measured gene expression, this method builds upon previous work in which genetic variants were utilized as causal anchors for gene expression [14, 15]. This allows for the identification of directed and unconfounded associations within observational data. Here, we adapt this method and identified 818 genes that affect DNA methylation using genomic, methylomic, and transcriptomic data in up to 4056 individuals [16, 17]. Many of these genes were previously unknown to be involved in the regulation of DNA methylation, thereby providing new targets for studies into epigenomic regulation, evaluation of the function of disease genes, and additional interpretation of epigenome-wide association studies.

## Results

### Identification of genes that affect DNA methylation

**Fig. 1:**
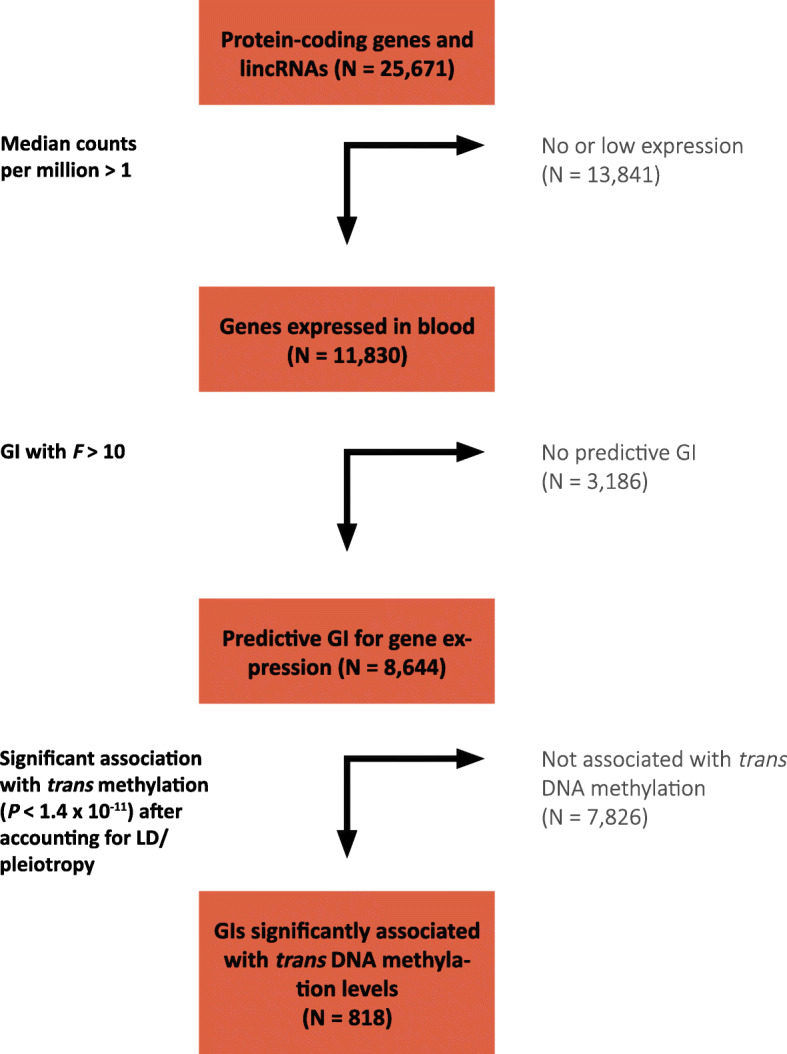
Flowchart showing the successive steps leading to the identification of 818 genes that affect DNA methylation *in trans*

In order to identify genes that affect DNA methylation, we employed an approach that consists of two parts. First, we identified predictive genetic variants for the expression of each gene in our data and aggregated these into single predictive scores termed genetic instruments (GIs) [13]. Second, we used these GIs as causal anchors to establish *directed* effects of gene expression on genome-wide DNA methylation levels, while ensuring that these associations were *specific* by accounting for linkage disequilibrium (LD) and pleiotropy among neighboring GIs (see Fig. 1 for an overview of the successive steps in the analysis).

To construct the genetic instruments, we used data on 3357 unrelated individuals with available genotype and RNAseq data derived from whole blood. We focused the analysis on 11,830 expressed genes (median counts per million > 1). In the training set (1/3 of the data, 1119 individuals), we obtained a GI for the expression of each gene, which consisted of 1 or more SNPs selected by applying LASSO regression to nearby genetic variants [18]. We corrected the expression data for age, sex, biobank, blood cell composition, and five principal components. We then assessed the predictive ability of the constructed GIs in a separate test set of 2238 individuals by predicting their gene expression values using the GIs derived in the training set. Of the 11,830 tested GIs, 8644 were sufficiently predictive of expression levels in the test set to serve as valid GIs (*F*-statistic > 10, median *R*^2^ = 0.04, Additional file 1: Table S1) [19].

Next, we tested for an association between all 8644 predictive GIs and genome-wide DNA methylation levels at 428,126 autosomal CpG sites *in trans* (> 10 Mb distance from the tested gene), using genotype and DNA methylation data (Illumina 450k array) derived from whole blood of 4056 unrelated individuals (3251 samples overlapped with RNAseq data). These associations were computed using linear regression, while correcting for age, sex, blood cell composition, biobank, and five principal components, and test statistics were corrected for bias and inflation [20]. These analyses resulted in *directed* associations between 2223 genes and 5284 CpGs (Bonferroni correction, *P* < 1.4 × 10^−11^; Additional file 2: Table S2). Although directed, the associations resulting from this analysis may not be specific for a single gene as linkage disequilibrium (LD) and/or pleiotropy may result in GIs that are predictive of multiple neighboring genes [13]. We therefore adjusted all significant GI-CpG pairs for all neighboring GIs (< 1 Mb) to account for correlation induced by LD/pleiotropy among neighboring genes. This enabled us to identify the specific gene in a region driving the directed association. Next, we removed genes with potential residual pleiotropic effects on the expression of neighboring significant genes (*F* > 5) (together, these two steps led to the removal of 1387 genes and 2844 CpGs; Additional file 3: Table S3). Finally, we excluded effects of long-range pleiotropy and LD (by rerunning the analysis for CpGs affected by multiple genes from the same chromosome, including all these genes in the model; removing 6 genes and 13 CpGs), and residual effects of white blood cell composition (by correcting for genetic variants known to be associated with WBC; removing 12 genes, 43 CpGs, Additional file 4: Fig. S1) [21, 22].

The final result of our step-wise analysis was a collection of 818 genes with directed and specific associations with DNA methylation levels of 2384 unique target CpGs *in trans* (Bonferroni correction, *P* < 1.4 × 10^−11^; (Additional file 5: Table S4). The target CpGs were located in 1915 distinct regions (consecutive probes within < 1 kb), and for genes affecting DNA methylation at more than 1 CpG site, on average 33% of the target CpGs were co-localized (< 1 kb) with at least one other target CpG (Additional file 6: Table S5).

The validity of these results was corroborated by a comparison with previous *trans*-methylation QTL studies in blood. Although not designed to infer genes that are specifically responsible for associations, such studies are expected to produce partly overlapping outcomes. We found that 1638 target CpGs identified in our study were reported in three previous independent *trans*-meQTL studies (OR = 103; *P* < 1 × 10^−32^) [23–25]. For the great majority of overlapping CpGs, the corresponding GI and *trans*-meQTL SNP were in close proximity (Additional file 4: Table S6, Additional file 7: Table S7, Additional file 8: Table S8, Additional file 9: Table S9).

We performed post hoc power analyses to assess the power we had to detect varying effect sizes for each gene tested (Additional file 4: Fig. S2 and Additional file 1: Table S1) [26]. In the uncorrected analysis (not corrected for neighboring GIs), we had > 0.8 power to detect effect sizes of 1 SD (1 standard deviation change in DNA methylation upon 1 standard deviation change in expression) for about 85% of the tested genes, and for about 50% of the genes (4475), we had > 0.8 power to detect effect sizes of 0.5 SD (Additional file 4: Fig. S2). Correcting for neighboring GIs is required to identify specific genes (instead of genomic regions with multiple correlated genes), but does so at the cost of reduced power. Correction left 5685 genes (compared to 7299) with power > 0.8 to detect effect sizes of 1 SD and left 3061 genes (compared to 4475) with > 0.8 power to detect effect sizes of 0.5 SD (Additional file 4: Fig. S2). This analysis shows that for the majority of tested genes, we were well-powered to detect large effects, and for over a third of the genes, we were well-powered to detect medium effect sizes. We included the explained variance and power across varying effect sizes for each gene in Additional file 1: Table S1.

### Function of genes that affect DNA methylation *in trans*

**Fig. 2:**
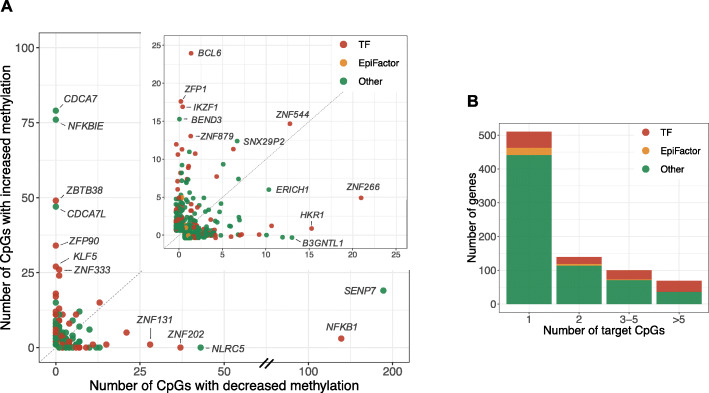
A considerable fraction of the identified genes (*N* = 308) affected multiple target CpGs *in trans*. **a** Each dot represents a gene with *trans* DNA methylation effects. The *x*-axis shows the number of affected target CpGs with decreased methylation levels upon increased gene expression, and the *y*-axis shows the number of affected target CpGs with increased methylation levels upon increased gene expression. The figure in the right upper corner is a zoomed-in version in which only genes that affect less than 25 CpG sites in either direction are displayed. **b** Bars represent the number of genes with either 1, 2, 3–5, or more than 5 target CpGs. The percentage of genes that are annotated as transcription factors increases with the number of target CpGs

As shown in Fig. 2, a considerable fraction (*N* = 308) of the identified genes affected multiple CpGs *in trans* (Additional file 6: Table S5). We observed that these genes, often consistently, either increased or decreased DNA methylation at their target CpGs (Fig. 2a). For 30 out of 37 genes that were associated with 10 or more CpGs, the direction of effect was significantly skewed towards increased (19 genes) or decreased (11 genes) methylation levels, respectively (binomial test, FDR < 0.05, Additional file 10: Table S10). We first considered two previously hypothesized molecular roles of the identified genes: transcription factors [27] and core epigenetic factors [6], which we will now discuss in more detail.

#### Transcription factors

**Fig. 3:**
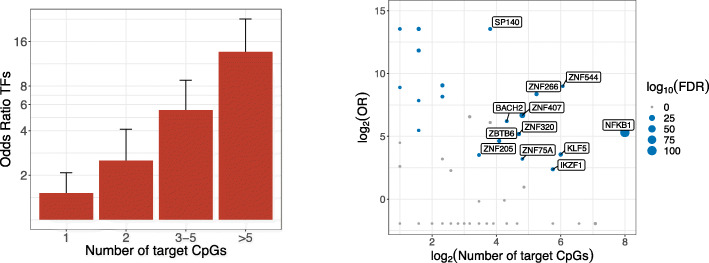
**a** Enrichment (odds ratio) for transcription factors among identified genes with either 1, 2, 3–5, or more than 5 target CpGs. Error bars represent 95% confidence intervals. **b** Transcription factor binding site enrichment; each dot represents a transcription factor, with on the *x*-axis the logarithm of the number of target CpGs for that transcription factor (at a gene-level significance level; *P* < 1.2 × 10^−7^), and on the *y*-axis the odds ratio for the enrichment of the target CpGs in its experimentally determined binding sites (ChIP-seq). The size of the dots represents the significance (FDR), and TFs for which the target CpGs were significantly enriched in its binding sites are colored in blue

We found that the identified genes (818) were enriched for transcription factors (TFs) (*N* = 127, odds ratio = 2.74, *P* = 3.1 × 10^−18^) using a manually curated list of TFs [27]. This enrichment was not explained by TFs having stronger genetic instruments; in fact, non-TFs had stronger instruments than TFs (*P* = 6.3 × 10^−8^; Additional file 4: Fig. S3). As shown in Fig. 3a, this enrichment was driven by TFs that were associated with multiple target CpGs, and there was a stronger TF enrichment with an increasing number of target CpGs. In total, 80 (63%) of the significant TFs in our data affected more than 1 CpG site, which was a significant enrichment compared to the non-TF genes (OR = 3.45, *P =* 3.1 × 10^−10^). We further found that the target CpGs of TFs frequently co-localized. For TFs affecting more than 1 CpG, on average, 45% of the target CpGs were co-localized with at least one other target CpG (< 1 kb), which was a significant enrichment compared to non-TFs (average non-TFs = 25%, OR = 2.5, *P* = 2.2 × 10^−21^). The majority of TFs either consistently increased or consistently decreased DNA methylation at their target CpGs: a significant skew in the direction of effect was present for 20 out of 23 TFs that were associated with at least 10 CpGs (6 consistently decreased, and 14 consistently increased DNA methylation at the target CpGs, respectively). TFs affecting the most CpGs included *NFKB1*, a key immune regulator (142 target CpGs; 127 regions, that is multiple CpGs spaced less than 1 kb); *ZBTB38*, a methyl-binding TF (49 target CpGs; 34 regions); and *ZNF202*, a zinc finger protein involved in lipid metabolism (37 target CpGs; 19 regions). One hundred out of the 127 (79%) TFs belonged to the C2H2 zinc finger family (odds ratio = 3.07, *P* = 5.2 × 10^−7^), of which the majority (*N* = 70) contained a KRAB domain. In line with the enrichment for TFs and zinc fingers, the gene set was overrepresented in the GO terms Nucleic Acid binding (*N* = 99, *P* = 1.1 × 10^−14^), DNA Binding (*N* = 114, *P* = 4.7 × 10^−9^), Metal Ion binding (*N* = 146, *P* = 1.4 × 10^−8^), and transcription factor activity (*N* = 73, *P =* 4.4 × 10^−8^) (Additional file 11: Table S11).

To assess whether TFs may affect DNA methylation directly (i.e., at their binding sites), we leveraged existing ChIP-seq data [28]. For each TF, we determined the overlap between the target CpGs (at a gene-level significant threshold: *P* < 1.2 × 10^−7^) and its experimentally determined binding sites as compared to a GC-content matched background. ChIP-seq data was available for 59 out of 110 TFs affecting multiple CpGs (at *P* < 1.2 × 10^−7^). For one third of these TFs (*N* = 20), target CpGs were significantly enriched for co-localization with their respective TF binding sites (FDR < 0.05; Fig. 3b, Additional file 12: Table S12).

#### Core epigenetic factors

Next, we compared our findings with a manually curated database of core epigenetic factors (EpiFactors) [6]. This database is mainly focused on the core enzymes that directly write, maintain, and/or establish epigenetic marks, but it also includes a few “borderline cases”, such as TFs that interact with epigenetic proteins. We found that 36 of the identified genes overlapped with genes in this database (odds ratio = 1.02, *P* > 0.05), of which 12 affected more than 1 CpG, which did not constitute a significant enrichment compared to the other genes affecting multiple CpGs (odds ratio = 0.82, *P* > 0.05)*.* Interestingly, the majority of the 36 genes encode proteins that target histone proteins (27 out of 36, OR = 1.13, *P* > 0.05). Another 7 genes were also annotated as TFs in the manually curated TF catalog [27]. The core epigenetic factors associated with most target CpGs include transcription factor *IKZF1* (positively associated with methylation at 17 target CpGs), histone demethylase *KDM5B* (positively associated with methylation at 7 target CpGs), and *BRD3*, which recognizes acetylated lysine residues on histones (positively associated with methylation at 5 target CpGs). The significant core epigenetic factors also included the DNA methyltransferase *DNMT3A*, which was associated with increased methylation at five target CpGs. Further exploration of potential *DNMT3A* targets indicated that the test statistics of *DNMT3A* were skewed towards increased DNA methylation levels, compatible with widespread but small effects (Additional file 4: Fig. S4). Of note, of the other main DNA methylation modifiers (*DNMT1*, *DNMT3B*, *TET1,2,3*), we had a sufficiently predictive GI for *DNMT1* only. However, both in the corrected and the uncorrected (for neighboring GIs) analyses, we did not find significant associations for this gene (Additional file 4: Fig. S4), although the statistical power of the uncorrected analysis was very similar to that of *DNMT3A* (Additional file 1: Table S1).

#### Other mechanisms underlying regulation of DNA methylation

Finally, the majority of the identified genes (*N* = 662) did not belong to the two a priori categories (TFs and core epigenetic factors; Fig. 2). A small fraction of these genes encodes proteins with DNA-binding properties (*N* = 24, OR = 0.91, *P* > 0.05). *BEND3*, for example, is a DNA-binding protein that was associated with increased methylation at 15 CpG sites. A previous study showed that BEND3 represses transcription by attracting the MBD3/NuRD complex that initiates histone deacetylation [29].

GO term enrichment analysis did not reveal significant functions underlying these genes. To explore possible biological functions among these genes, we provide case studies below for the five genes from this set that were associated with the most target CpGs: *SENP7* (189 target CpGs), *CDCA7* (79 target CpGs), *NFKBIE* (76 target CpGs), *CDCA7L* (47 target CpGs), and *NLRC5* (43 target CpGs).

#### NFKBIE

**Fig. 4:**
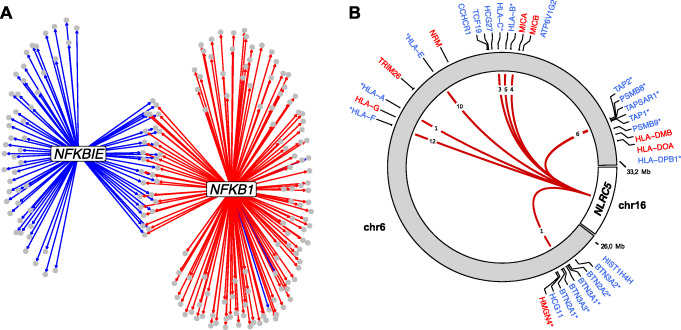
**a** Network for transcription factor *NFKB1* and its inhibitor *NFKBIE*. Gray circles indicate target CpGs, and arrows represent directed associations (i.e., association between GI and DNA methylation levels). Blue lines indicate a positive association between gene expression and DNA methylation levels; red lines indicate a negative association between gene expression levels and DNA methylation levels. **b** *NLRC5* (chromosome 16) was associated with decreased DNA methylation levels at multiple (*N* = 43) CpG sites in the classical and extended MHC region (chromosome 6). Red lines indicate a negative association between *NLRC5* expression levels and DNA methylation levels. The numbers displayed in the lines indicate how many target CpGs the line represents. Gene labels are displayed if one or more target CpGs were associated with the expression of these genes. Blue gene symbols refer to genes negatively correlated with target CpG methylation (implying upregulation by *NLRC5*), and vice versa for red labels. Asterisks indicate that the GI corresponding to *NLRC5* was also (positively) associated with this gene

The *NFKBIE* gene encodes IκBε which is an inhibitor of NFκB, a transcription factor that plays a fundamental role in the regulation of the immune response [30, 31]. IκBε binds to components of NFκB and retains it in the cytoplasm, thereby preventing it from activating genes in the nucleus. Consistent with the previous interpretation of a *trans*-methylation QTL effect [16], increased expression of *NFKB1* was associated with genome-wide loss of DNA methylation. In contrast, increased expression of *NFKBIE* resulted in *higher* methylation levels at 76 CpG sites across the genome (70 regions). In line with its role as NFκB inhibitor, a substantial number of its target CpGs (28) overlap with NFκB’s target CpGs and show opposite effects (Fig. 4a). To further characterize the target CpGs, we overlapped the CpGs with trait-associated probes included in EWASdb [32] (results for all genes are included in Additional file 13: Table S13). The target CpGs were enriched for CpGs associated with obesity/BMI, consistent with the role of NFκB in obesity-related inflammation [33].

#### NLRC5

Increased expression of *NLRC5* was associated with decreased methylation levels at 43 CpG sites (11 regions), which were all located in either the classical or the extended MHC region [34]. NLRC5 is a known activator of MHC class I genes [35], and in line with this, the methylation levels of most target CpGs (*N* = 36) were negatively associated with the expression levels of one or more neighboring MHC genes (Fig. 4b/Additional file 14: Table S14, Additional file 15: Table S15). Furthermore, the GI corresponding to *NLRC5* was positively associated with the expression of 16 of these genes. NLRC5 itself does not contain a DNA-binding domain; instead, it has been shown to affect transcription by cooperating with a multi-protein complex that is assembled on the MHC class I promoter [35]. Interestingly, NLRC5 acts as a platform for enzymes that open chromatin by histone acetylation and/or demethylation of histone H3, indicating that decreased DNA methylation may be a consequence of altered chromatin state. In line with the role of NLRC5 in immune response, the target CpGs of *NLRC5* were enriched for CpGs that were previously associated with immune-related disorders (including auto-immune disorders primary Sjögren’s syndrome and mixed connective tissue disease and sTNFR2 levels; Additional file 13: Table S13) [32].

#### SENP7

The gene with the largest number of detected target CpGs was *SENP7*. It was associated with decreased methylation levels at 189 target CpGs (87 regions) and with increased methylation levels at 19 target CpGs (12 regions). The majority (86%) of the target CpGs were located on the q-arm of chromosome 19. For most of these CpGs (92%), the DNA methylation levels were associated with the expression levels of one or more nearby zinc fingers (Additional file 16: Table S16, Additional file 17: Table S17), consistent with a previous gene network analysis [13]. Although SENP7 has no DNA-binding properties, previous research has shown that it exerts its effect through deSUMOylation of the chromatin repressor KAP1 [36]. KAP1 can act as a scaffold for various heterochromatin-inducing factors, and there is emerging evidence that KAP1 is directly involved in regulating DNA methylation [37, 38]. SENP7 could therefore affect DNA methylation through its interaction with KAP1. We further characterized *SENP7* target CpGs by overlapping the CpGs with trait-related probes and found an enrichment for Werner syndrome-associated CpGs [32]. Interestingly, the Werner syndrome gene product is modified by SUMO [39, 40] and may therefore be related to SENP7’s function as SUMO protease.

#### CDCA7

**Fig. 5:**
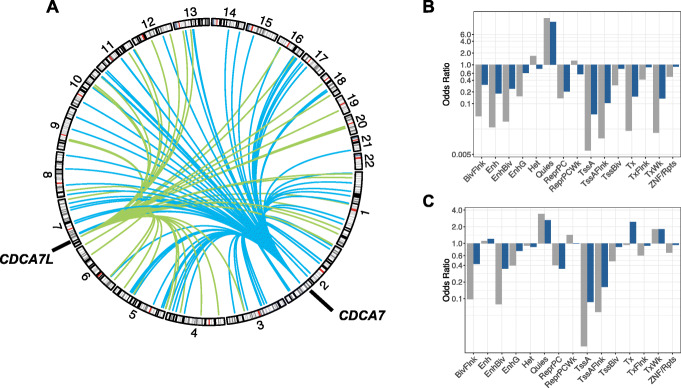
**a** *CDCA7* (located on chromosome 2) and *CDCA7L* (located on chromosome 7) both affect genome-wide DNA methylation levels. Blue lines indicate a positive association between *CDCA7* expression and *trans* DNA methylation levels. Green lines indicate a positive association between *CDCA7L* expression levels and *trans* DNA methylation levels. **b**, **c** Over- or underrepresentation of target CpGs in predicted chromatin states for **b** *CDCA7* and **c** *CDCA7L*. Blue bars represent enrichment of CpGs that are significant at a genome-wide significance level (*P* < 1.4 × 10^−11^), and gray bars represent enrichment of CpGs that are significant at a gene-level significance level (*P* < 1.2 × 10^−7^). BivFlnk, flanking bivalent TSS/enhancer; Enh, enhancer; EnhBiv, bivalent enhancer; EnhG, genic enhancer; Het, heterochromatin; Quies, quiescent; ReprPC, repressed polycomb; ReprPCWk, weak repressed polycomb; TssA, active TSS; TssAFlnk, flanking active TSS; TssBiv, bivalent/poised TSS; Tx, strong transcription; TxFlnk, weak transcription; ZNF/Rpts, ZNF genes and repeats

Mutations in *CDCA7* have been shown to cause ICF syndrome, a rare primary immunodeficiency characterized by epigenetic abnormalities [41]. Previous research showed that *CDCA7*-mutated ICF patients show decreased DNA methylation levels at pericentromeric repeats and heterochromatin regions, and similarly, *CDCA7* depletion in mouse embryonic fibroblasts leads to decreased DNA methylation at centromeric repeats [41, 42]. In line with this prior work, increased expression of *CDCA7* was associated with increased methylation levels at 79 CpG sites (79 regions) that were distributed across chromosomes (Fig. 5a) and were enriched in low-activity regions (e.g., quiescent states; Fig. 5b) [43]. In addition, the target CpGs were enriched in repeat sequences as defined by the UCSC RepeatMasker (odds ratio 2.13, *P* = 0.006) [44]. A volcano plot showed that the test statistics of *CDCA7* were highly skewed towards positive effects, suggesting that *CDCA7* has widespread effects on DNA methylation (Additional file 4: Fig. S5a).

#### CDCA7L

*CDCA7L* is a paralog of *CDCA7*, and similarly, its increased expression was associated with a genome-wide increase of DNA methylation levels (47 CpG sites, 47 regions; Fig. 5a and Additional file 4: Fig. S5b). *CDCA7L*’s target CpGs did not overlap with those of *CDCA7*; however, they did show a similar genomic distribution and were enriched in inactive regions (Fig. 5c), although enrichment at repeat regions as defined in the UCSC RepeatMasker was reduced (OR = 1.59, *P* > 0.05). Interestingly, previous research has shown that the risk allele of the genetic variant most highly associated with multiple myeloma (rs4487645) was associated with increased *CDCA7L* expression [45]. Our GI for *CDCA7L* consisted of 5 SNPs, of which one (rs17361667) was in strong LD (*r*^*2*^ = 0.7) with the risk variant rs4487645. If the risk variant indeed exerts its pathogenic effect through an effect on *CDCA7L* expression, *CDCA7L*’s effects on DNA methylation might be involved in the pathogenesis of multiple myeloma. Moreover, our multi-SNP GI was a stronger predictor of *CDCA7L* expression (*F* = 171) as compared with rs4487645 (*F* = 60) and may therefore be useful in gaining more insight into the role of *CDCA7L* in multiple myeloma.

## Discussion

Our genome-wide analysis, utilizing genetic instruments for gene expression, identified 818 genes that affect distant DNA methylation levels in blood and provide insights into the principles of epigenetic regulation. Our results highlight a role of TFs. TFs were overrepresented among the identified genes and either consistently increased or decreased DNA methylation at their target CpGs. For multiple TFs, we could show that the associated target CpGs also preferentially co-localized with experimentally determined binding sites (examples include *NFKB1*, *ZNF544*, *KLF5*, *ZNF266*, and *IKZF1*). In line with these findings, previous studies suggest that TFs can regulate the acquisition and loss of DNA methylation at their binding sites [8, 9, 46]. For example, several TFs have been shown to recruit DNMTs to their binding sites, thereby causing de novo DNA methylation [47–50]. Conversely, TFs have been indicated to protect against the acquisition of DNA methylation by blocking de novo methylation or by interacting with TET proteins [10, 11, 16, 50]. We identified TFs with a previously unrecognized role in the regulation of DNA methylation (e.g., *ZNF202*, *ZNF131* and *ZFP90*) and provided support for the presumed role of TFs as previously implicated by post hoc interpretation of results from meQTL mapping (*NFKB1* and *ZBTB38*) [16]. Interestingly, many of the TFs we identified were members of the C2H2 zinc finger family, which is in line with previous *trans*-meQTL findings [24, 25]. The majority of the identified zinc finger TFs contained a Krüppel-associated box (KRAB) domain, which has been implicated in epigenetic silencing through the recruitment of KAP1 to its binding sites. KAP1 subsequently recruits proteins that establish heterochromatin such as the NuRD complex and possibly DNMTs, thereby causing de novo methylation [51–53]. Although we found 8 KRAB-ZFs with at least 10 target CpGs that were significantly skewed towards increased methylation, four were associated with decreased methylation. A possible explanation is that not all KRAB-ZFs act via KAP1. For example, the KRAB-ZF *ZNF202*, which was negatively associated with 37 target CpGs, contains a SCAN domain that prevents the binding of KAP1 [54]. Overall, our systematic genome-wide analysis identifies novel epigenetic regulatory functions for TFs, significantly expands upon TFs that were previously implicated in DNA methylation regulation, and identifies the direction of the effect on DNA methylation.

Exploration of the genes that do not encode TFs revealed several potential mechanisms through which genes may affect DNA methylation. First, several of the genes encode proteins with DNA-binding properties, which might recruit or block the DNA methylation machinery in a similar way to TFs. *BEND3*, for example, encodes a DNA-binding protein that attracts the chromatin remodeling NuRD complex to its binding sites [29]. Second, exploration of the top five non-DNA-binding genes with the highest number of associated target CpGs suggests that protein-protein interactions are among the possible mechanisms. The mechanisms include post-translational regulation (*NFKBIE* encodes for IκBε which retains NF-κB in the cytoplasm [30]), post-translational modification (SENP7: deSUMOylates the repressor KAP1 [13]), and recruitment of epigenetic proteins to specific target sites through association with a DNA-binding protein (NLRC5 associates with a protein complex in MHC-I region [35]). Third, a subset of the identified genes overlapped with genes in a database that focuses on the core epigenetic regulators (i.e., the main enzymes that write or erase epigenetic marks, such as DNMTs and histone acetyltransferases) [6]. Among these was *DNMT3A*, for which we identified five target CpGs. Finally, we note that the majority of genes that were previously identified as core epigenetic factors (EpiFactor database) are histone modifiers [6]. This suggests that changes in DNA methylation may be secondary to altered chromatin conformation. This idea is further supported by discussed examples such as *IKZF1*, *BEND3*, and *NLRC5*, which are thought to attract histone-modifying complexes to their binding sites [29, 35, 55]. Thus, our findings underpin earlier observations that DNA methylation and histone modifications are interdependent [56].

Conceptually, our method has similarities with previous applications that used genetic variation to infer associations between gene expression and phenotypic outcomes [14, 15]. To the best of our knowledge, these methods have not been used to investigate directed associations between gene expression and DNA methylation. A key feature of our implementation is that it explicitly controls for LD/pleiotropy among neighboring genes and hence yields directed associations that are specific for a single gene [13]. Indeed, we observed that, if LD/pleiotropy is not considered, 60% of genes seemingly associated with DNA methylation in fact involved unspecific effects.

Our method is designed to identify genes with a directed and specific association with DNA methylation. This results in critical differences in interpretation of results as compared with *trans*-methylation QTL studies. *Trans*-methylation QTL studies report on genetic variants associated with distant DNA methylation. Since genetic variants are often not readily interpretable, a mix of post hoc analyses, including evaluation of nearest genes and cis-expression QTL mapping, are commonly performed to link genetic variants to genes [16, 23–25]. However, these analyses do not control for LD/pleiotropy, and as we showed here, this severely limits the possibility of correctly implicating the specific gene involved.

An additional advantage of focusing on genes as functionally interpretable units instead of genetic variants is that this increases power by reducing multiple testing (10 thousands genes vs. millions of genetic variants). Power of our gene-focused approach is further increased by using multi-SNP instruments which are usually better predictors of gene expression than single SNPs [13, 14].

We should, however, also note limitations of our method. First, our method does not distinguish between mechanistically direct or indirect effects of gene expression on DNA methylation. An example in this regard is *NFKBIE*, which affects DNA methylation through inhibition of the transcription factor NFκB. Similarly, TFs could affect DNA methylation indirectly through the regulation of another gene. We note, however, that CpGs affected by TFs commonly co-localized with their respective binding sites, favoring the interpretation of a direct effect. Second, the main assumption in our analysis is that the genetic instruments affect DNA methylation through their effect on gene expression. Although we systematically considered LD/pleiotropy among neighboring genes, the genetic instruments may have pleiotropic effects on unmeasured genes. In addition, although trained to capture variation in gene expression, genetic instruments may inadvertently be associated with *trans*-DNA methylation through other mechanisms than expression such as interchromosomal contacts [16]. In principal, further studies could investigate such pleiotropic effects using statistical methods including Egger’s regression and heterogeneity tests [57]. These methods, however, rely on multiple independent variants which are scarce for gene expression, since most predictive variants are located near the gene and are therefore often not independent because of LD. Third, although we intended to provide a genome-wide resource of genes that affect DNA methylation, we had to limit our scope to genes that had a sufficiently predictive genetic instrument. Fourth, the statistical power of our method is limited because genetic instruments generally explain a relatively small proportion of the variation in expression of their corresponding gene (Additional file 1: Table S1; Additional file 4: Fig. S2). We further note that for significant genes, limited power will often underestimate the true number of CpG sites affected by the respective gene. An additional factor reducing power is the correction for nearby GIs, which is required to obtain specific associations but at the same time leads to the loss of true effects. Hence, we expect that the genes affecting distant DNA methylation we report on here can be significantly expanded on by applying our method to datasets obtained using more comprehensive DNA methylation profiling assays than the 450k array, to larger sample numbers (see power analyses in Additional file 4: Fig. S6), and, in particular, to other tissues than blood.

We envision multiple applications of our findings. First, we identified many genes that were previously unknown to be involved in the regulation of DNA methylation. Importantly, the genes were enriched for transcription factors that, in turn, commonly affected DNA methylation at their binding sites, thereby providing new targets for studies into epigenomic regulation. Second, epigenetic dysregulation is a hallmark of many diseases, and in line with this, mutations in genes regulating the epigenome are increasingly reported to be involved in Mendelian disease [1]. We found that 200 out of the 818 genes we implicated in the regulation of DNA methylation were known disease genes (OMIM; Additional file 18: Table S18) [58]. Our results may aid in elucidating downstream effects of these disease genes. An interesting example in this regard is *CDCA7L*, which we found to affect DNA methylation throughout the genome in a similar fashion as *CDCA7*. Mutations in *CDCA7* lead to the ICF syndrome, a syndrome characterized by hypomethylation in pericentric repeats [41]. Since we found that *CDCA7L* has similar effects on DNA methylation, it may be hypothesized that mutations in *CDAC7L* lead to similar phenotypes. Finally, altered DNA methylation patterns have been reported for many environmental exposures and traits using epigenome-wide association studies (EWAS). However, it often remains unclear how these patterns are established [46]. The target CpGs identified in our analyses can aid in interpreting EWAS results and may point to the signal transduction pathways relaying external and internal stimuli to the methylome. To illustrate this point, we overlapped the identified target CpGs with existing EWAS results (Additional file 13: Table S13) and found that target CpGs of several genes were enriched for trait-associated CpGs, including Werner syndrome (*SENP7*, a SUMO peptidase; SUMO modifies the Werner syndrome gene product [39, 40]), auto-immune diseases and inflammatory markers (*NLRC5*, a key regulator of MHC class I-dependent immune response [35]), and obesity/BMI (*NFKBIE* and *NFKB1*; NFκB is a central regulator of inflammatory response, including metabolism-related inflammation [33]).

## Conclusions

We present a collection of genes for which we provide strong evidence that they affect DNA methylation levels in blood. Our results add to the increasing evidence that transcription factors are involved in shaping the methylome, and we demonstrate that our results can provide insight into the various mechanisms through which DNA methylation is regulated (e.g., post-translation modification and secondary effects of chromatin conformation). We believe these results can guide follow-up studies into epigenetic regulation, the role of these regulatory genes in disease, and the pathways mediating differential methylation as detected in EWASs.

## Methods

### Cohorts

The Biobank-based Integrative Omics Study (BIOS) Consortium comprises six Dutch biobanks: Cohort on Diabetes and Atherosclerosis Maastricht (CODAM) [59], LifeLines-DEEP (LLD) [60], Leiden Longevity Study (LLS) [61], Netherlands Twin Registry (NTR) [62, 63], Rotterdam Study (RS) [64], and Prospective ALS Study Netherlands (PAN) [65]. Data used in this study consists of 4162 unrelated individuals for which genotype data was available. For 4056 of these individuals, DNA methylation data was available, and for 3357 individuals, RNA-sequencing data was available. Genotype data, DNA methylation data, and gene expression data were measured in whole blood. In addition, sex, age, and cell counts were obtained. The Human Genotyping facility (HuGe-F, Erasmus MC, Rotterdam, The Netherlands, http://www.glimdna.org) generated the methylation and RNA-sequencing data.

### Genotype data

Genotype data was generated for each cohort individually. Details on the methods used can be found in the individual papers—CODAM: [66], LLD: [60], LLS: [67], NTR: [68], RS: [64], and PAN: [65]. The genotype data were harmonized towards the Genome of the Netherlands (GoNL) using Genotype Harmonizer [69] and subsequently imputed per cohort using MaCH [70] with the Haplotype Reference Consortium panel [71]. Per cohort, SNPs with *R*^2^ < 0.3 and call rate < 0.95 were removed, and VCFtools [72] was used to remove SNPs with Hardy-Weinberg equilibrium *P* value < 10^−4^. After merging the cohorts, SNPs with minor allele frequency < 0.01 were removed. These imputation and filtering steps resulted in 7,568,624 SNPs that passed quality control in each of the datasets.

### Gene expression data

A detailed description regarding generation and processing of the gene expression data can be found elsewhere [17]. Briefly, total RNA from whole blood was deprived of globin using Ambion’s GLOBIN clear kit and subsequently processed for sequencing using Illumina’s Truseq version 2 library preparation kit. Paired-end sequencing of 2 × 50 bp was performed using Illumina’s Hiseq2000, pooling 10 samples per lane. Finally, read sets per sample were generated using CASAVA, retaining only reads passing Illumina’s Chastity Filter for further processing. Data were generated by the Human Genotyping facility (HuGe-F) of Erasmus MC (The Netherlands). Initial QC was performed using FastQC (v0.10.1), removal of adaptors was performed using cutadapt (v1.1) [73], and Sickle (v1.2) [74] was used to trim low-quality ends of the reads (minimum length 25, minimum quality 20). The sequencing reads were mapped to human genome (HG19) using STAR (v2.3.0e) [75].

To avoid reference mapping bias, all GoNL SNPs (http://www.nlgenome.nl/?page_id=9) with MAF > 0.01 in the reference genome were masked with N. Read pairs with at most 8 mismatches, mapping to as most 5 positions, were used.

Gene expression quantification was determined using base counts [17]. The gene definitions used for quantification were based on Ensembl version 71, with the extension that regions with overlapping exons were treated as separate genes and reads mapping within these overlapping parts did not count towards expression of the normal genes.

For data analysis, we used the log counts per million (CPM). We restricted the analysis to protein-coding genes and lincRNAs (long intergenic non-coding RNAs) that were at least moderately expressed (median CPM ≥ 1). This resulted in 11,475 protein-coding genes and 355 lincRNAs that were used for further analysis. To reduce the influence of possible outliers, we transformed the data using rank-based inverse normal transformation within each cohort [76–78].

### DNA methylation data

The Zymo EZ DNA methylation kit (Zymo Research, Irvine, CA, USA) was used to bisulfite-convert 500 ng of genomic DNA, and 4 μl of bisulfite-converted DNA was measured on the Illumina HumanMethylation450 array using the manufacturer’s protocol (Illumina, San Diego, CA, USA). Preprocessing and normalization of the data were done as described in the DNAmArray workflow (https://molepi.github.io/DNAmArray_workflow/). In brief, IDAT files were read using the *minfi* [79], while sample-level quality control (QC) was performed using *MethylAid* [80]. Filtering of individual measurements was based on detection *P* value (*P* < 0.01), number of beads available (≤ 2), or zero values for signal intensity. Normalization was done using functional normalization [81] as implemented in *minfi* [79], using five principal components extracted using the control probes for normalization. All samples or probes with more than 5% of their values missing were removed.

#### Probe filtering

Since it has been shown that the Dutch population contains population-specific variation, we identified genetic variants that overlap with probes using release 5 variant data from the GoNL project (https://molgenis26.target.rug.nl/downloads/gonl_public/variants/release5/) [82]. This data contains 20.4 million SNVs and 1.1 million short INDELs (1–20 bp) obtained from WGS data from 498 unrelated Dutch individuals. *BCFtools* was used to extract the INFO files from the GoNL VCF files [83]. The genomic coordinates were stored in *GRanges* format in R [84]; for deletions, we used the length of the deletion to define the start and end coordinates of the deletion. The *findOverlaps* function in the *GenomicRanges* package was used to identify variants that were located in the SBE site for type I probes (the SBE site coincides with the C-nucleotide in type II probes), CpG site, or within 5 bases of the 3′-end of the probe. Since not all SNPs at SBE sites of type I probes cause a color-channel switch, only SNPs that cause a color-channel switch (A/G, G/T, and C/G SNPs for reverse strand probes and C/T, C/A, and C/G SNPs for forward strand probes) and INDELs overlapping the SBE were flagged for removal. A list of all SNPs and short INDELs that overlap with 450K probes is available from https://github.com/molepi/DNAmArray.

We identified 15,724 probes that contained one or more variants with MAF > 0.01 in the SBE site (causing a color-channel switch), CpG site, or within 5 bases of the 3′-end and excluded these probes for further analyses. In addition, we removed probes with a non-unique mapping and non-unique 3′ nested subsequences of at least 30 bases as recommended by Zhou et al. [85]. In total, this led to the removal of 41,674 probes. Finally, we removed all probes on the sex chromosomes.

The final dataset consisted of 4056 samples and 428,126 probes. To reduce the influence of possible outliers, we transformed the data using rank-based inverse normal transformation within each cohort, similar to the RNAseq data.

Proper data linkage of SNP, RNAseq, and DNA methylation array data within individuals was verified using the *omicsPrint* package [86].

### Imputation of missing covariates

A fraction of the samples had missing data for the phenotype measures used in subsequent analyses (white blood cell proportions, age, and sex).

#### Overview missing data

White blood cell counts (neutrophils, eosinophils, lymphocytes, monocytes, and basophils) were measured as part of the complete blood cell count. Complete cell count measurements were missing for 35% of the RNAseq samples and 44% of the DNA methylation samples. Reported age and sex were missing for 1.5% of the RNAseq samples and 18% of the DNA methylation samples.

#### Imputation

Since DNA methylation and RNAseq data are informative for age, sex, and white blood cell composition [87–90], we used the data to impute these variables. Missing observations were imputed separately for the RNAseq and DNA methylation data because there is incomplete overlap between the datasets. Missing observations in the measured white blood cell counts (WBCC) were imputed using the R package *pls*, adjusting for reported age and sex, as described earlier (https://molepi.github.io/DNAmArray_workflow/05_Predict.html) [20]. For missing age and sex measurements, we compared the performance of the elastic net, LASSO, ridge, and pls methods. To evaluate the performance of these models, the data was randomly split into a train set (2/3) and a test set (1/3). This procedure was repeated 25 times, each time calculating the accuracy in the test set (mean squared error for age and *F*_1_-score for sex). The above algorithm was performed using varying numbers of input variables (50 to 10,000), where the input variables were selected based on their correlation with the outcome. The model and number of input variables that resulted in the best average accuracy in the test sets were selected to impute missing data. The average correlation between predicted and reported age in the tests sets was 0.98 for the DNA methylation data and 0.92 for the RNAseq data. Sex was almost perfectly predicted (accuracy ≈ 0.995) in both DNA methylation and RNAseq data.

### Constructing a local genetic instrument for gene expression

We constructed a genetic instrument (GI) for the expression of each gene using nearby genetic variants. We split the genotype and RNAseq data in a training set (one third of all samples, *N* = 1119) and a test set (two thirds of all samples, *N* = 2238), making sure all cohorts and both sexes were equally represented within each set. In the train set, we built a GI for the expression of each gene by employing a two-step approach in which LASSO regression is used for variable selection and coefficient estimation [18]. We previously reported that LASSO performs better (BLUP, BSLMM) or similar (elastic net) compared to other methods to create GIs [13].

The number of variables chosen by LASSO is generally large and potentially includes noise variables [91]. A two-step approach can overcome this problem, where LASSO is first used for variable selection and is then used again on the selected variables for coefficient estimation. In detail, for each gene, we performed the following procedure:

1. LASSO is performed in the training set to select nearby genetic variants (within the gene or within 100 kb of the gene’s transcription start site (TSS) or transcription end site (TES)) that are predictive of the expression of the respective gene. Fivefold cross-validation was used to find the penalization parameter *λ* that minimizes the mean squared error (MSE).
2. LASSO is performed in the training set on the remaining genetic variants. In order to select the most parsimonious model without losing accuracy, we used the “one-standard error rule” to select the largest penalization parameter *λ* that is within 1 standard error of the minimum with the constraint that at least one SNP has a non-zero coefficient [92]. We then calculated the genetic instrument as the sum of dosages of each SNP multiplied by their effect sizes:
  $$ {GI}_j=\boldsymbol{D}{\beta}_j $$

where *GI*_*j*_ is a vector of predicted expression levels for gene *j*, ***D*** is a matrix with dosage values for the nearby genetic variants of gene *j*, and *β*_*j*_ is a vector of coefficients as determined in the second LASSO step described above.

In both LASSO steps, we included known covariates (age, sex, cohort, and white blood cell composition) and the first five principal components derived from the RNAseq data in the LASSO model, because the inclusion of covariates that explain variation will generally lead to increased precision of the SNP coefficients [93]. These covariates were left unpenalized, ensuring that their coefficient is always non-zero.

We evaluated the predictive performance of the genetic instruments in the test set. We employed analysis of variance (ANOVA) to evaluate the added predictive power of the GI over the covariates, as reflected by the *F*-statistic. Genetic instruments with an *F*-statistic > 10 were considered valid instruments [19].

### Testing for *trans* effects

Using linear regression, we tested for an association between each GI *j* and the DNA methylation levels *k* at CpGs *in trans* (> 10 Mb):

$$ {\boldsymbol{DNAm}}_{\boldsymbol{k}}={\boldsymbol{GI}}_{\boldsymbol{j}}{\varphi}_j+\boldsymbol{C}\boldsymbol{\beta } +\varepsilon $$

where we test for the significance of the regression coefficient *φ*_*j*_, and ***C*** represents a covariate matrix including sampling age, sex, cohort, white blood cell composition, and five principal components. We used the Bioconductor package *bacon* to correct for inflation and/or bias in the test statistics [20] and corrected for multiple testing using the Bonferroni correction (8644 × 428,126 tests, *P* < 1.4 × 10^−11^). A two-step approach was used to account for LD/pleiotropy within the obtained results (Additional file 4: Fig. S7). First, we corrected all GI-CpG pairs for nearby GIs (within 1 Mb of the gene’s TSS/TES) using linear regression:

$$ {\boldsymbol{DNAm}}_{\boldsymbol{k}}={\boldsymbol{G}\boldsymbol{I}}_{\boldsymbol{j}}{\varphi}_j+\boldsymbol{C}\boldsymbol{\beta } +{\boldsymbol{G}}_{\boldsymbol{j}}\boldsymbol{\gamma} +\varepsilon $$

where we test for the significance of the regression coefficient *φ*_*j*_; ***C*** represents a covariate matrix including sampling age, sex, cohort, white blood cell composition, and five principal components; and ***G***_***j***_ represents a matrix with GIs of genes neighboring (< 1 Mb) index gene *j*. Genes for which the corresponding GI was highly correlated with one or more neighboring GIs (*r* > 0.95) were excluded from further analyses. To prevent collinearity, we pruned the neighboring GIs that were included in the model using the *findCorrelation* function in the *caret* R package using a correlation cutoff of 0.95 [94]. Second, among the GIs that remained significant, we tested for residual pleiotropic effects that were not captured by the correction for nearby GIs. For each GI, we evaluated the added predictive power over the covariates and neighboring GIs on the expression corresponding to nearby significant GIs. We excluded GIs that shared target CpGs with a neighboring significant GI (at a gene-level Bonferroni level, *P <* 1.2 × 10^−7^) and were at least weakly predictive of the expression of that gene (*F* > 5).

### Power analyses

We calculated statistical power to detect associations at a two-sided *α* of 1.4 × 10^−11^ based on the proportion of variance in gene expression explained by the genetic instruments, the sample size, and varying hypothetical effect sizes [26]. We evaluated the statistical power for both the uncorrected analysis (not including nearby genetic instruments) and the corrected analysis (including nearby genetic instruments, < 1 Mb). For the corrected analysis, we calculated power using the proportion of variance in gene expression explained taking into account the neighboring GIs (partial *R*^2^).

### Enrichment analyses

#### Gene set enrichment

Gene set enrichment was performed for GO molecular functions using DAVID [95], where all genes with a predictive GI (*F* > 10) were used as background. Fisher’s exact test was used to test for enrichment of transcription factors [27] and epigenetic factors [6].

#### Chromatin state enrichment

Chromatin state segments were downloaded from the Epigenomics Roadmap for all blood subtypes [43]. CpGs were annotated to different segments based on the most frequent occurring feature in the various blood cell subtypes. Repeat sequences were downloaded from the UCSC table browser [44]. Enrichment tests for chromatin state segments and repeat sequences were performed using Fisher’s exact test.

#### Transcription factor binding site enrichment

We obtained transcription factor ChIP-seq peaks called with the MACS2 software from the GTRD database (http://gtrd.biouml.org/), which contains uniformly processed ChIP-seq data from ENCODE and the Sequence Read Archive (SRA) [28, 96–98]. For 59 out of the 110 identified transcription factors associated with multiple CpGs (2 or more), at least 1 ChIP-seq experiment was available. For each TF, we overlapped target CpG locations (at a gene-level significant threshold, *P* < 1.2 × 10^−7^) and its experimentally determined binding sites (ChIP-seq peaks). If multiple experiments were available for a specific TF, we determined the overlap per experiment. The HOMER software was used to generate a background set for each TF with the same GC-content distribution as the target CpGs (100,000 regions) [99]. We performed Fisher’s exact test to determine whether the target CpGs overlapped with ChIP-seq peaks more often than the background regions. We employed a two-step approach to account for multiple testing, where first the Simes procedure was used to control for multiple experiments available per TF (since they are expected to be correlated), and second the Benjamini-Hochberg procedure was used to control the FDR among the tested TFs [100].

#### EWAS enrichments

Blood-related EWASs were downloaded from EWASdb (https://bigd.big.ac.cn/ewas/downloads) [32]. For each gene, we overlapped target CpGs (at a gene-level significant threshold, *P* < 1.2 × 10^−7^) with CpGs associated with each trait included in the EWAS database, and performed Fisher’s exact test to determine whether the target CpGs overlapped more often with trait-related CpGs than a background consisting of all probes included in the database. We limited the analysis to traits associated with < 10,000 CpGs.

### Association with *trans* expression levels

For several examples, we tested whether the target CpGs were associated with nearby gene expression and/or if the GI corresponding to the index gene was associated with the expression levels of genes near its target CpGs. We tested for an association between the target CpGs and the expression of nearby genes (< 250 kb) using linear regression. Age, sex, cohort, white blood cell composition, and 10 principal components (first five PCs derived from gene expression data, and first five PCs derived from DNA methylation data) were included as covariates. Similarly, to test whether the index GI was associated with the expression of genes near the target CpGs, we tested for an association between the GI and the expression of nearby genes (< 250 kb) using linear regression. Age, sex, cohort, and white blood cell compositions were included as covariates. In both analyses, we used *bacon* to correct for bias and inflation in the test statistics and adjusted for multiple correction using the Bonferroni correction [20].

## Supplementary information

**Additional file 1: Table S1.** The genetic instruments (GIs) and their predictive power as measured by the F-statistic and partial R^2^. This table also includes the power to detect various effect sizes for both the uncorrected and corrected analysis (for neighboring GIs).

**Additional file 2: Table S2.** Significant associations (*P* < 1.4 x 10^-11^) between GIs and *trans* CpGs, without taking LD/pleiotropy among neighboring genes into account.

**Additional file 3: Table S3.** Associations between GIs and *trans* CpGs that remained significant (*P* < 1.4 x 10^-11^) after taking LD/pleiotropy among neighboring genes into account.

**Additional file 4: Table S6.** Comparison with previous *trans*-methylation QTL studies in blood. **Fig. S1.** Test-statistics before and after adjustment for SNPs associated with white blood cell counts. **Fig. S2.** Power analyses. **Fig. S3.** Explained variance of genetic instruments for TFs and non-TFs. **Fig. S4.** Volcano plots for *DNMT3A* and *DNMT1.* **Fig. S5.** Volcano plots for *CDCA7* and *CDCA7L.* **Fig. S6.** Increase in statistical power with larger sample sizes. **Fig. S7.** Diagram showing the presumed relations between genetic instruments, expression and DNA methylation.

**Additional file 5: Table S4.** Associations between GIs and *trans* CpGs that remained significant (*P* < 1.4 x 10^-11^) after taking LD/pleiotropy among neighboring genes into account and that were insensitive to additional tests of the underlying assumptions of the analyses. This is the final dataset that was used for further analyses.

**Additional file 6: Table S5.** The 818 identified genes, their classification (TF/EpiFactor/Other), and for each gene the number of positive/negative associations, the number of regions affected (consisting of consecutive probes within <1Kb) and the number of regions of various sizes affected.

**Additional file 7: Table S7.** Target CpGs that overlap with *trans*-meQTL CpGs identified by Lemire et al.

**Additional file 8: Table S8.** Target CpGs that overlap with *trans*-meQTL CpGs identified by Gaunt et al.

**Additional file 9: Table S9.** Target CpGs that overlap with *trans*-meQTL CpGs identified by Huan et al.

**Additional file 10: Table S10.** Genes that associate with at least 10 target CpGs and the number of positive and negative associations for each gene (significance level from binomial test).

**Additional file 11: Table S11.** GO molecular functions terms (DAVID).

**Additional file 12: Table S12.** Transcription factor binding site enrichments, includes the overlap between target CpGs and ChIP-seq peaks for each experiment, the overlap between the background regions and the ChIP-seq peaks, and the enrichment statistics (Fisher’s exact test).

**Additional file 13: Table S13.** Significant overlap between target CpGs and trait-associated CpGs (EWASdb, Liu et al.).

**Additional file 14: Table S14.** Significant associations between the DNA methylation levels of *NLRC5* target CpGs and the expression of nearby genes (<250Kb).

**Additional file 15: Table S15.** Significant associations between the GI corresponding to *NLRC5* and the expression of genes near its target CpGs (<250Kb).

**Additional file 16: Table S16.** Significant associations between the DNA methylation levels of *SENP7* target CpGs and the expression of nearby genes (<250Kb).

**Additional file 17: Table S17.** Significant associations between the GI corresponding to *SENP7* and the expression of genes near its target CpGs (<250Kb).

**Additional file 18: Table S18**. OMIM phenotypes linked to the identified genes.

**Additional file 19.** Review history.

## Data Availability

Data are available from the European Genome-Phenome Archive (EGAC00001000277) [101]. Scripts for the main analyses are available at: https://github.com/pjhop/DNAmRegulators [102]. Complete results can be browsed in and downloaded from the BBMRI Atlas (http://bbmri.researchlumc.nl/atlas/).

## References

[1] Bjornsson HT (2015). The Mendelian disorders of the epigenetic machinery. Genome Res.

[2] Dor Y, Cedar H (2018). Principles of DNA methylation and their implications for biology and medicine. Lancet.

[3] Sen P, Shah PP, Nativio R, Berger SL (2016). Epigenetic mechanisms of longevity and aging. Cell.

[4] Zentner GE, Henikoff S (2013). Regulation of nucleosome dynamics by histone modifications. Nat Struct Mol Biol.

[5] Schübeler D (2015). Function and information content of DNA methylation. Nature.

[6] Medvedeva YA, Lennartsson A, Ehsani R, Kulakovskiy IV, Vorontsov IE, Panahandeh P (2015). EpiFactors: a comprehensive database of human epigenetic factors and complexes. Database.

[7] Shen H, Laird PW (2013). Interplay between the cancer genome and epigenome. Cell.

[8] Marchal C, Miotto B (2015). Emerging concept in DNA methylation: role of transcription factors in shaping DNA methylation patterns: transcription factors in DNA methylation. J Cell Physiol.

[9] Blattler A, Farnham PJ (2013). Cross-talk between site-specific transcription factors and DNA methylation states. J Biol Chem.

[10] Wang Y, Xiao M, Chen X, Chen L, Xu Y, Lv L (2015). WT1 recruits TET2 to regulate its target gene expression and suppress leukemia cell proliferation. Mol Cell.

[11] Stadler MB, Murr R, Burger L, Ivanek R, Lienert F, Schöler A (2011). DNA-binding factors shape the mouse methylome at distal regulatory regions. Nature..

[12] Daxinger L, Harten SK, Oey H, Epp T (2013). An ENU mutagenesis screen identifies novel and known genes involved in epigenetic processes in the mouse. Genome Biol.

[13] Luijk R, Dekkers KF, van Iterson M, Arindrarto W, Claringbould A, Hop P (2018). Genome-wide identification of directed gene networks using large-scale population genomics data. Nat Commun.

[14] Gamazon ER, Wheeler HE, Shah KP, Mozaffari SV, Aquino-Michaels K, Carroll RJ (2015). A gene-based association method for mapping traits using reference transcriptome data. Nat Genet.

[15] Gusev A, Ko A, Shi H, Bhatia G, Chung W, Penninx BWJH (2016). Integrative approaches for large-scale transcriptome-wide association studies. Nat Genet.

[16] Bonder MJ, Luijk R, Zhernakova DV, Moed M, Deelen P, Vermaat M (2017). Disease variants alter transcription factor levels and methylation of their binding sites. Nat Genet.

[17] Zhernakova DV, Deelen P, Vermaat M, van Iterson M, van Galen M, Arindrarto W (2017). Identification of context-dependent expression quantitative trait loci in whole blood. Nat Genet.

[18] Tibshirani R (1996). Regression shrinkage and selection via the lasso. J R Stat Soc Ser B.

[19] Staiger D, Stock JH (1997). Instrumental variables regression with weak instruments. Econometrica.

[20] van Iterson M, van Zwet EW, Heijmans BT. Controlling bias and inflation in epigenome- and transcriptome-wide association studies using the empirical null distribution. Genome Biol. 2017;18 http://genomebiology.biomedcentral.com/articles/10.1186/s13059-016-1131-9. Accessed 2 Feb 2017. 10.1186/s13059-016-1131-9 PMC5273857 28129774

[21] Orrù V, Steri M, Sole G, Sidore C, Virdis F, Dei M (2013). Genetic variants regulating immune cell levels in health and disease. Cell.

[22] Roederer M, Quaye L, Mangino M, Beddall MH, Mahnke Y, Chattopadhyay P (2015). The genetic architecture of the human immune system: a bioresource for autoimmunity and disease pathogenesis. Cell.

[23] Gaunt TR, Shihab HA, Hemani G, Min JL, Woodward G, Lyttleton O (2016). Systematic identification of genetic influences on methylation across the human life course. Genome Biol.

[24] Lemire M, Zaidi SHE, Ban M, Ge B, Aïssi D, Germain M (2015). Long-range epigenetic regulation is conferred by genetic variation located at thousands of independent loci. Nat Commun.

[25] Huan T, Joehanes R, Song C, Peng F, Guo Y, Mendelson M (2019). Genome-wide identification of DNA methylation QTLs in whole blood highlights pathways for cardiovascular disease. Nat Commun.

[26] Brion M-JA, Shakhbazov K, Visscher PM (2013). Calculating statistical power in Mendelian randomization studies. Int J Epidemiol.

[27] Lambert SA, Jolma A, Campitelli LF, Das PK, Yin Y, Albu M (2018). The human transcription factors. Cell.

[28] Yevshin I, Sharipov R, Kolmykov S, Kondrakhin Y, Kolpakov F (2019). GTRD: a database on gene transcription regulation—2019 update. Nucleic Acids Res.

[29] Saksouk N, Barth TK, Ziegler-Birling C, Olova N, Nowak A, Rey E (2014). Redundant mechanisms to form silent chromatin at pericentromeric regions rely on BEND3 and DNA methylation. Mol Cell.

[30] Bonizzi G, Karin M (2004). The two NF-κB activation pathways and their role in innate and adaptive immunity. Trends Immunol.

[31] Liu T, Zhang L, Joo D, Sun S-C (2017). NF-κB signaling in inflammation. Signal Transduct Target Ther.

[32] Liu D, Zhao L, Wang Z, Zhou X, Fan X, Li Y (2019). EWASdb: epigenome-wide association study database. Nucleic Acids Res.

[33] Tornatore L, Thotakura AK, Bennett J, Moretti M, Franzoso G (2012). The nuclear factor kappa B signaling pathway: integrating metabolism with inflammation. Trends Cell Biol.

[34] Mungall AJ, Palmer SA, Sims SK, Edwards CA, Ashurst JL, Wilming L, et al. The DNA sequence and analysis of human chromosome 6. Nature. 2003;425:805–11. 10.1038/nature02055 14574404

[35] Kobayashi KS, van den Elsen PJ (2012). NLRC5: a key regulator of MHC class I-dependent immune responses. Nat Rev Immunol.

[36] Garvin AJ, Densham RM, Blair-Reid SA, Pratt KM, Stone HR, Weekes D (2013). The deSUMOylase SENP7 promotes chromatin relaxation for homologous recombination DNA repair. EMBO Rep.

[37] Quenneville S, Verde G, Corsinotti A, Kapopoulou A, Jakobsson J, Offner S (2011). In embryonic stem cells, ZFP57/KAP1 recognize a methylated hexanucleotide to affect chromatin and DNA methylation of imprinting control regions. Mol Cell.

[38] Zuo X, Sheng J, Lau H-T, McDonald CM, Andrade M, Cullen DE (2012). Zinc finger protein ZFP57 requires its co-factor to recruit DNA methyltransferases and maintains DNA methylation imprint in embryonic stem cells via its transcriptional repression domain. J Biol Chem.

[39] Kawabe Y, Seki M, Seki T, Wang W-S, Imamura O, Furuichi Y (2000). Covalent modification of the Werner’s syndrome gene product with the ubiquitin-related protein, SUMO-1. J Biol Chem.

[40] Yannone SM, Roy S, Chan DW, Murphy MB, Huang S, Campisi J (2001). Werner syndrome protein is regulated and phosphorylated by DNA-dependent protein kinase. J Biol Chem.

[41] Thijssen PE, Ito Y, Grillo G, Wang J, Velasco G, Nitta H (2015). Mutations in CDCA7 and HELLS cause immunodeficiency–centromeric instability–facial anomalies syndrome. Nat Commun.

[42] Velasco G, Grillo G, Touleimat N, Ferry L, Ivkovic I, Ribierre F (2018). Comparative methylome analysis of ICF patients identifies heterochromatin loci that require ZBTB24, CDCA7 and HELLS for their methylated state. Hum Mol Genet.

[43] Kundaje A, Meuleman W, Ernst J, Bilenky M, Yen A, Heravi-Moussavi A (2015). Integrative analysis of 111 reference human epigenomes. Nature.

[44] Karolchik D (2004). The UCSC Table Browser data retrieval tool. Nucleic Acids Res.

[45] Li N, Johnson DC, Weinhold N, Studd JB, Orlando G, Mirabella F (2016). Multiple myeloma risk variant at 7p15.3 creates an IRF4-binding site and interferes with CDCA7L expression. Nat Commun.

[46] Zhu H, Wang G, Qian J (2016). Transcription factors as readers and effectors of DNA methylation. Nat Rev Genet.

[47] Di Croce L, Raker V, Corsaro M, Fazi F, Fanelli M, Faretta M (2002). Methyltransferase recruitment and DNA hypermethylation of target promoters by an oncogenic transcription factor. Science.

[48] Brenner C, Deplus R, Didelot C, Loriot A, Viré E, De Smet C (2005). Myc represses transcription through recruitment of DNA methyltransferase corepressor. EMBO J.

[49] Velasco G, Hube F, Rollin J, Neuillet D, Philippe C, Bouzinba-Segard H (2010). Dnmt3b recruitment through E2F6 transcriptional repressor mediates germ-line gene silencing in murine somatic tissues. Proc Natl Acad Sci.

[50] de la Rica L, Rodríguez-Ubreva J, García M, Islam AB, Urquiza JM, Hernando H (2013). PU. 1 target genes undergo Tet2-coupled demethylation and DNMT3b-mediated methylation in monocyte-to-osteoclast differentiation. Genome Biol.

[51] Meylan S, Groner AC, Ambrosini G, Malani N, Quenneville S, Zangger N (2011). A gene-rich, transcriptionally active environment and the pre-deposition of repressive marks are predictive of susceptibility to KRAB/KAP1-mediated silencing. BMC Genomics.

[52] Groner AC, Meylan S, Ciuffi A, Zangger N, Ambrosini G, Dénervaud N (2010). KRAB–zinc finger proteins and KAP1 can mediate long-range transcriptional repression through heterochromatin spreading. PLoS Genet.

[53] Iyengar S, Ivanov AV, Jin VX, Rauscher FJ, Farnham PJ (2011). Functional analysis of KAP1 genomic recruitment. Mol Cell Biol.

[54] Lupo A, Cesaro E, Montano G, Zurlo D, Izzo P, Costanzo P (2013). KRAB-zinc finger proteins: a repressor family displaying multiple biological functions. Curr Genomics.

[55] Oestreich KJ, Weinmann AS (2012). Ikaros changes the face of NuRD remodeling. Nat Immunol.

[56] Cedar H, Bergman Y (2009). Linking DNA methylation and histone modification: patterns and paradigms. Nat Rev Genet.

[57] Burgess S, Bowden J, Fall T, Ingelsson E, Thompson SG (2017). Sensitivity analyses for robust causal inference from Mendelian randomization analyses with multiple genetic variants. Epidemiology.

[58] McKusick-Nathans Institute of Genetic Medicine, Johns Hopkins. Online Mendelian Inheritance in Man, OMIM®. 2020. https://omim.org/.

[59] van Greevenbroek MMJ, Jacobs M, van der Kallen CJH, Vermeulen VMM-J, Jansen EHJM, Schalkwijk CG (2011). The cross-sectional association between insulin resistance and circulating complement C3 is partly explained by plasma alanine aminotransferase, independent of central obesity and general inflammation (the CODAM study). Eur J Clin Investig.

[60] Tigchelaar EF, Zhernakova A, Dekens JA, Hermes G, Baranska A, Mujagic Z (2015). Cohort profile: LifeLines DEEP, a prospective, general population cohort study in the northern Netherlands: study design and baseline characteristics. BMJ Open.

[61] Schoenmaker M, de Craen AJ, de Meijer PH, Beekman M, Blauw GJ, Slagboom PE (2006). Evidence of genetic enrichment for exceptional survival using a family approach: the Leiden Longevity Study. Eur J Hum Genet EJHG.

[62] Boomsma DI, Vink JM, Van Beijsterveldt TC, de Geus EJ, Beem AL, Mulder EJ (2002). Netherlands Twin Register: a focus on longitudinal research. Twin Res Hum Genet.

[63] Willemsen G, Vink JM, Abdellaoui A, den Braber A, van Beek JHDA, Draisma HHM (2013). The Adult Netherlands Twin Register: twenty-five years of survey and biological data collection. Twin Res Hum Genet.

[64] Hofman A, Murad SD, van Duijn CM, Franco OH, Goedegebure A, Arfan Ikram M (2013). The Rotterdam study: 2014 objectives and design update. Eur J Epidemiol.

[65] Huisman MHB, de Jong SW, van Doormaal PTC, Weinreich SS, Schelhaas HJ, van der Kooi AJ (2011). Population based epidemiology of amyotrophic lateral sclerosis using capture-recapture methodology. J Neurol Neurosurg Psychiatry.

[66] van Dam RM, Boer JM, Feskens EJ, Seidell JC (2001). Parental history of diabetes modifies the association between abdominal adiposity and hyperglycemia. Diabetes Care.

[67] Deelen J, Beekman M, Uh H-W, Broer L, Ayers KL, Tan Q (2014). Genome-wide association meta-analysis of human longevity identifies a novel locus conferring survival beyond 90 years of age. Hum Mol Genet.

[68] Lin BD, Willemsen G, Abdellaoui A, Bartels M, Ehli EA, Davies GE (2016). The genetic overlap between hair and eye color. Twin Res Hum Genet.

[69] Deelen P, Bonder MJ, van der Velde KJ, Westra H-J, Winder E, Hendriksen D (2014). Genotype harmonizer: automatic strand alignment and format conversion for genotype data integration. BMC Res Notes.

[70] Li Y, Willer CJ, Ding J, Scheet P, Abecasis GR (2010). MaCH: using sequence and genotype data to estimate haplotypes and unobserved genotypes. Genet Epidemiol.

[71] McCarthy S, Das S, Kretzschmar W, Delaneau O, Wood AR, Teumer A (2016). A reference panel of 64,976 haplotypes for genotype imputation. Nat Genet.

[72] Danecek P, Auton A, Abecasis G, Albers CA, Banks E, DePristo MA (2011). The variant call format and VCFtools. Bioinformatics..

[73] Martin M (2011). Cutadapt removes adapter sequences from high-throughput sequencing reads. EMBnet J.

[74] Joshi NA, Fass JN (2011). Sickle: a sliding-window, adaptive, quality-based trimming tool for FastQ files (version 1.33).

[75] Dobin A, Davis CA, Schlesinger F, Drenkow J, Zaleski C, Jha S (2013). STAR: ultrafast universal RNA-seq aligner. Bioinformatics..

[76] Wang K, Huang J. A score-statistic approach for the mapping of quantitative-trait loci with sibships of arbitrary size. Am J Hum Genet. 2002;70:412–24. 10.1086/338659 PMC384916 11791211

[77] Pain O, Dudbridge F, Ronald A (2018). Are your covariates under control? How normalization can re-introduce covariate effects. Eur J Hum Genet.

[78] Peng B, Yu RK, DeHoff KL, Amos CI (2007). Normalizing a large number of quantitative traits using empirical normal quantile transformation. BMC Proc.

[79] Aryee MJ, Jaffe AE, Corrada-Bravo H, Ladd-Acosta C, Feinberg AP, Hansen KD (2014). Minfi: a flexible and comprehensive Bioconductor package for the analysis of Infinium DNA methylation microarrays. Bioinformatics.

[80] van Iterson M, Tobi EW, Slieker RC, den Hollander W, Luijk R, Slagboom PE (2014). MethylAid: visual and interactive quality control of large Illumina 450k datasets. Bioinformatics.

[81] Fortin J-P, Labbe A, Lemire M, Zanke BW, Hudson TJ, Fertig EJ (2014). Functional normalization of 450k methylation array data improves replication in large cancer studies. Genome Biol.

[82] Francioli LC, Menelaou A, Pulit SL, van Dijk F, Palamara PF, Elbers CC (2014). Whole-genome sequence variation, population structure and demographic history of the Dutch population. Nat Genet.

[83] Li H, Handsaker B, Wysoker A, Fennell T, Ruan J, Homer N (2009). The Sequence Alignment/Map format and SAMtools. Bioinformatics.

[84] Lawrence M, Huber W, Pagès H, Aboyoun P, Carlson M, Gentleman R (2013). Software for computing and annotating genomic ranges. PLoS Comput Biol.

[85] Zhou W, Laird PW, Shen H (2017). Comprehensive characterization, annotation and innovative use of Infinium DNA methylation BeadChip probes. Nucleic Acids Res.

[86] van Iterson M, Cats D, Hop P, Heijmans BT, BIOS consortium (2018). omicsPrint: detection of data linkage errors in multiple omics studies. Bioinformatics.

[87] Horvath S. DNA methylation age of human tissues and cell types. Genome Biol. 2013;14. 10.1186/gb-2013-14-10-r115 PMC4015143 24138928

[88] Houseman EA, Accomando WP, Koestler DC, Christensen BC, Marsit CJ, Nelson HH (2012). DNA methylation arrays as surrogate measures of cell mixture distribution. BMC Bioinformatics.

[89] Abbas AR, Wolslegel K, Seshasayee D, Modrusan Z, Clark HF (2009). Deconvolution of blood microarray data identifies cellular activation patterns in systemic lupus erythematosus. PLoS One.

[90] Peters MJ, Joehanes R, Pilling LC, Schurmann C, Conneely KN, Powell J (2015). The transcriptional landscape of age in human peripheral blood. Nat Commun.

[91] Meinshausen N (2007). Relaxed lasso. Comput Stat Data Anal.

[92] James G, Witten D, Hastie T, Tibshirani R. An introduction to statistical learning. 7th ed. New York: Springer Texts in Statistics; 2013.

[93] Burgess S, Thompson SG, CRP CHD Genetics Collaboration (2011). Avoiding bias from weak instruments in Mendelian randomization studies. Int J Epidemiol.

[94] Kuhn M. Building predictive models in R using the caret package. J Stat Softw. 2008;28.

[95] Huang DW, Sherman BT, Lempicki RA (2008). Systematic and integrative analysis of large gene lists using DAVID bioinformatics resources. Nat Protoc.

[96] Zhang Y, Liu T, Meyer CA, Eeckhoute J, Johnson DS, Bernstein BE (2008). Model-based analysis of ChIP-Seq (MACS). Genome Biol.

[97] Landt SG, Marinov GK, Kundaje A, Kheradpour P, Pauli F, Batzoglou S (2012). ChIP-seq guidelines and practices of the ENCODE and modENCODE consortia. Genome Res.

[98] Kodama Y, Shumway M, Leinonen R, on behalf of the International Nucleotide Sequence Database Collaboration (2012). The sequence read archive: explosive growth of sequencing data. Nucleic Acids Res.

[99] Heinz S, Benner C, Spann N, Bertolino E, Lin YC, Laslo P (2010). Simple combinations of lineage-determining transcription factors prime cis-regulatory elements required for macrophage and B cell identities. Mol Cell.

[100] Luijk R, Goeman JJ, Slagboom EP, Heijmans BT, van Zwet EW (2015). An alternative approach to multiple testing for methylation QTL mapping reduces the proportion of falsely identified CpGs. Bioinformatics.

[101] Heijmans BT, Hoen PAC ’t, van Meurs J, Boomsma DI, Pool R, van Dongen J, et al. The BIOS Consortium: Biobank-based Integrative Omics Studies. EGA. https://ega-archive.org/dacs/EGAC00001000277 (2020).

[102] Hop PJ, Luijk R, van Zwet EW, Heijmans BT. Genome-wide identification of genes regulating DNA methylation using genetic anchors for causal inference. Github. https://github.com/pjhop/DNAmRegulators (2020). 10.1186/s13059-020-02114-z PMC7453518 32859263

