# Supplementary material for: Genome-wide identification of genes regulating DNA methylation using genetic anchors for causal inference
Source: Genome Biol. 2020 Aug 28;21:220. doi: 10.1186/s13059-020-02114-z (PMC7453518; doi:10.1186/s13059-020-02114-z)
Supplement: Supplementary file 4 — Additional file 4: Table S6. Comparison with previous trans-methylation QTL studies in blood. Fig. S1. Test-statistics before and after adjustment for SNPs associated with white blood cell counts. Fig. S2. Power analyses. Fig. S3. Explained variance of genetic instruments for TFs and non-TFs. Fig. S4. Volcano plots for DNMT3A and DNMT1. Fig. S5. Volcano plots for CDCA7 and CDCA7L. Fig. S6. Increase in statistical power with larger sample sizes. Fig. S7. Diagram showing the presumed relations between genetic instruments, expression and DNA methylation. [file 13059_2020_2114_MOESM4_ESM.pdf]

Table S6

Study	Number of overlapping CpGs	Number of overlapping CpGs with corresponding GI and *trans*-meQTL SNP in close proximity (<1Mb)	Enrichment
Lemire *et al.* (2015)	504	489	OR=97; $P < 1 \times 10^{-32}$
Gaunt *et al.* (2016)	413	408	OR=116; $P < 1 \times 10^{-32}$
Huan *et al.* (2019)	1,520	1,482	OR=88; $P < 1 \times 10^{-32}$
Total	1,628	1,589	OR=103; $P < 1 \times 10^{-32}$

Table S6. Comparison with previous *trans*-methylation QTL studies in blood.
We found a considerable overlap (N = 1,628) between the target CpGs identified in our study (N = 2,384) and the CpGs identified in three independent *trans*-meQTL studies (Huan *et al.* Nature Communications (2019), Gaunt *et al.* Genome Biology (2016), Lemire *et al*. Nature Communications (2015)) (table S7-9). For the great majority of overlapping CpGs, the corresponding GI and *trans*-meQTL SNP were in close proximity (N = 1,589).

Fig. S1

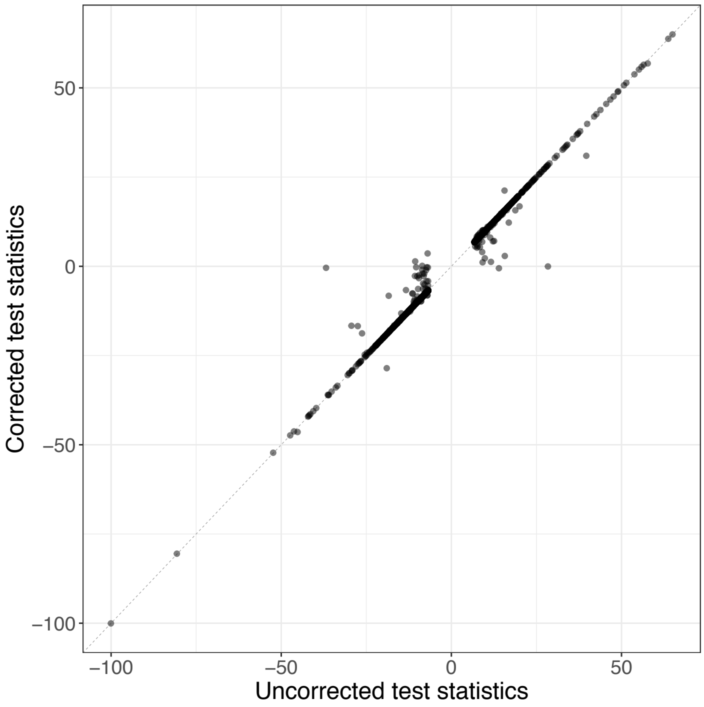

Fig. S1. Test-statistics before and after adjustment for SNPs associated with white blood cell counts.

Test-statistics of the 2,633 GI-CpG pairs before (x-axis) and after (y-axis) adjustment for nearby (<1Mb) SNPs associated with white blood cell composition. 48 GI-CpG pairs were insignificant after adjustment.

Fig. S2

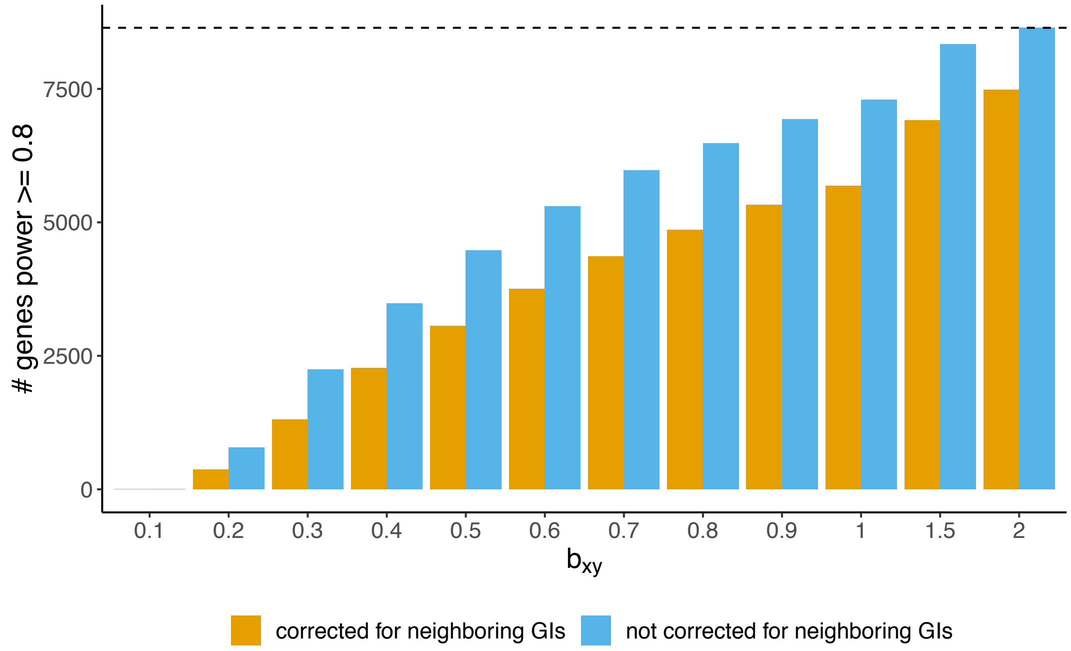

Fig. S2. Power analyses.

Number of genes for which we had >0.8 power to detect effects across varying effect sizes both with and without correcting for neighboring GIs. b_{xy} (x-axis) represents the effect of gene expression on DNA methylation (1SD change in DNA methylation upon 1SD change in gene expression). b_{xy} is calculated as $b_{xy} = b_{zy}/b_{zx}$, where b_{zy} is the effect of the GI on DNA methylation, and b_{zx} is the effect of the GI on gene expression.

Fig. S3

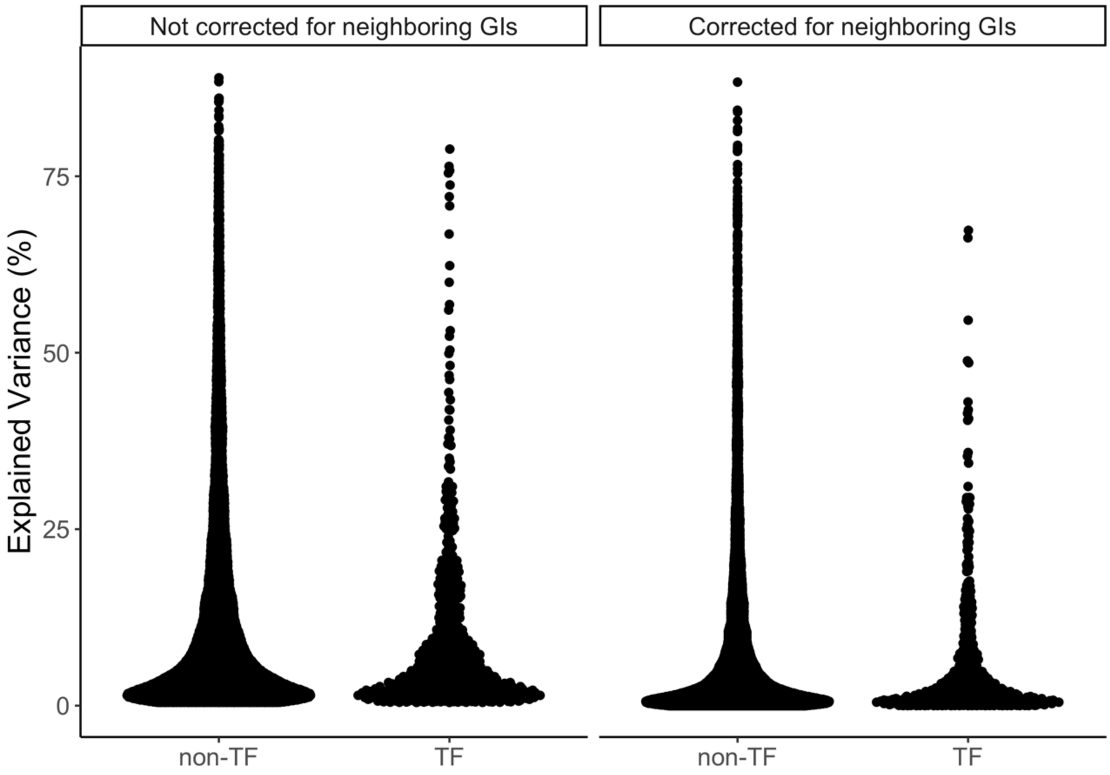

Fig. S3. Explained variance of genetic instruments for TFs and non-TFs.
Explained variance for all tested genes, split by TFs and non-TFs. The left panel shows the explained variance not corrected for neighboring GIs, the right panel shows the explained variance corrected for neighboring GIs.

Fig. S4

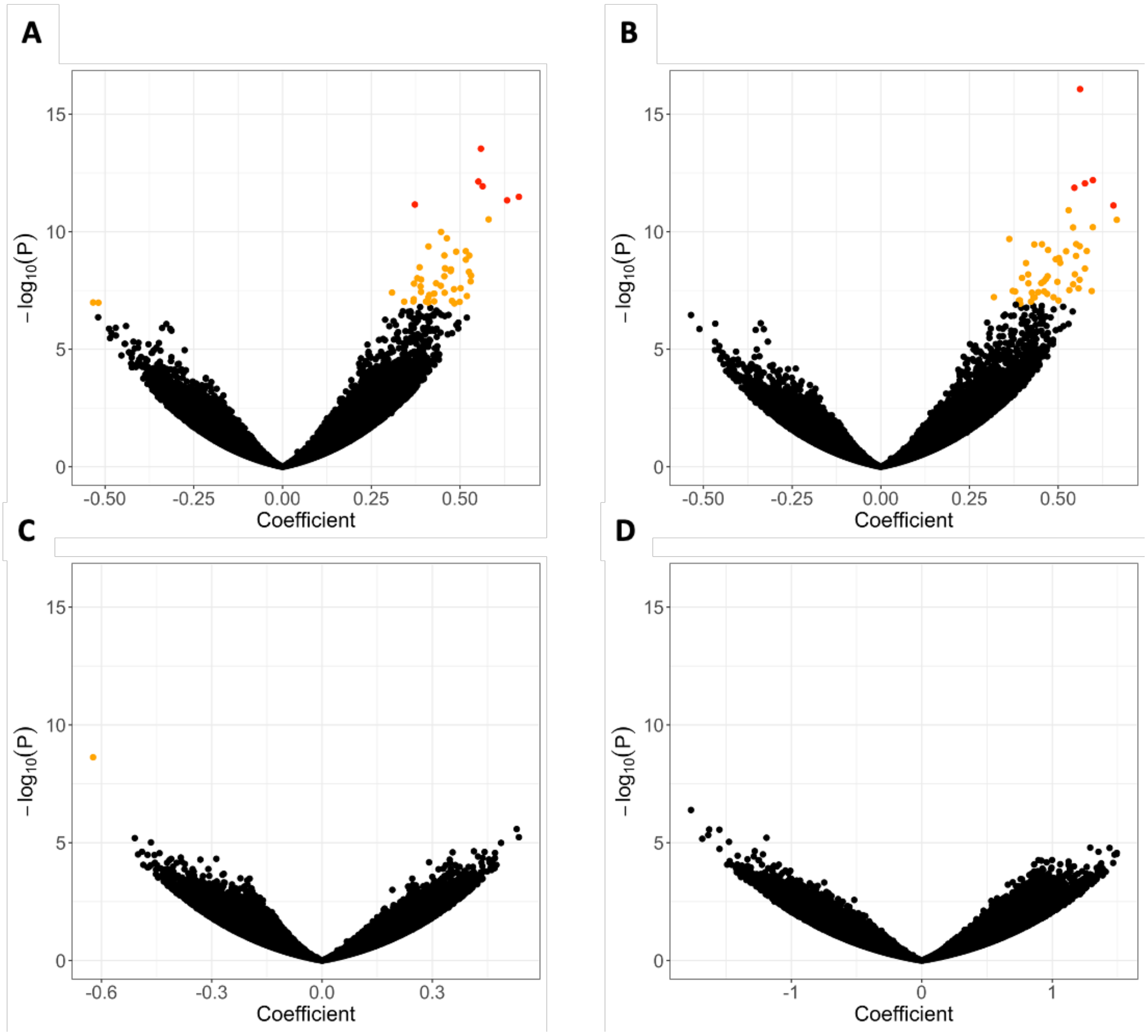

Fig. S4. Volcano plots for *DNMT3A* and *DNMT1.*
Volcano plot showing the estimated coefficients (x-axis) and $-\log_{10}$(*P*-values) (y-axis) of the genetic instrument corresponding to (A and B) *DNMT3A* and (C and D) *DNMT1* on *trans* DNA methylation levels. A and C represent the test-statistics from the analysis uncorrected for neighboring GIs for *DNMT3A* and *DNMT1* respectively. B and D represent the test-statistics from the analysis corrected for neighboring GIs for *DNMT3A* and *DNMT1* respectively.
Red dots indicate CpG-sites that are significant at $P < 1.4 \times 10^{-11}$ and orange dots indicate CpG-sites that are significant at $P < 1.2 \times 10^{-7}$ (corresponding to a gene-level look up, 428,126 tests).

Fig. S5

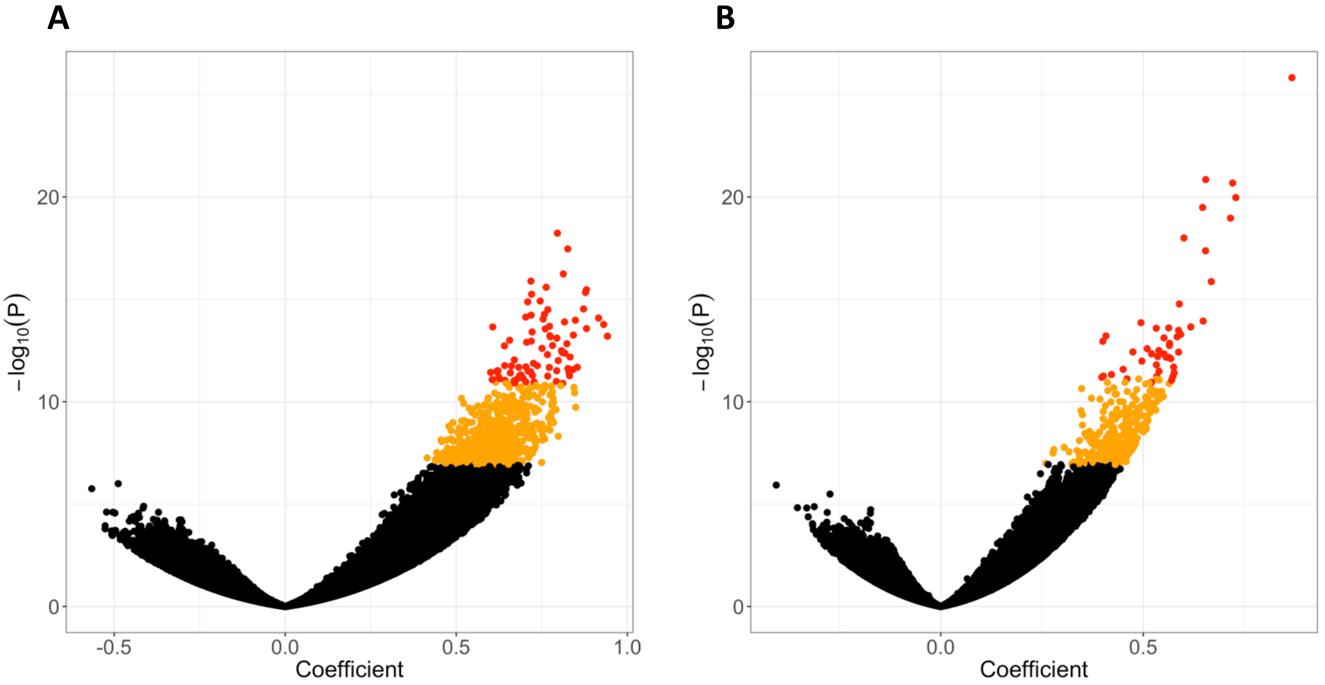

Fig. S5. Volcano plots for *CDCA7* and *CDCA7L.*
Volcano plot showing the estimated coefficients (x-axis) and $-\log_{10}$(*P*-values) (y-axis) of the genetic instrument corresponding to (a) *CDCA7* and (b) *CDCA7L* on *trans* DNA methylation levels. Red dots indicate CpG-sites that are significant at $P < 1.4 \times 10^{-11}$ and orange dots indicate CpG-sites that are significant at $P < 1.2 \times 10^{-7}$ (corresponding to a gene-level look up, 428,126 tests).

Fig. S6

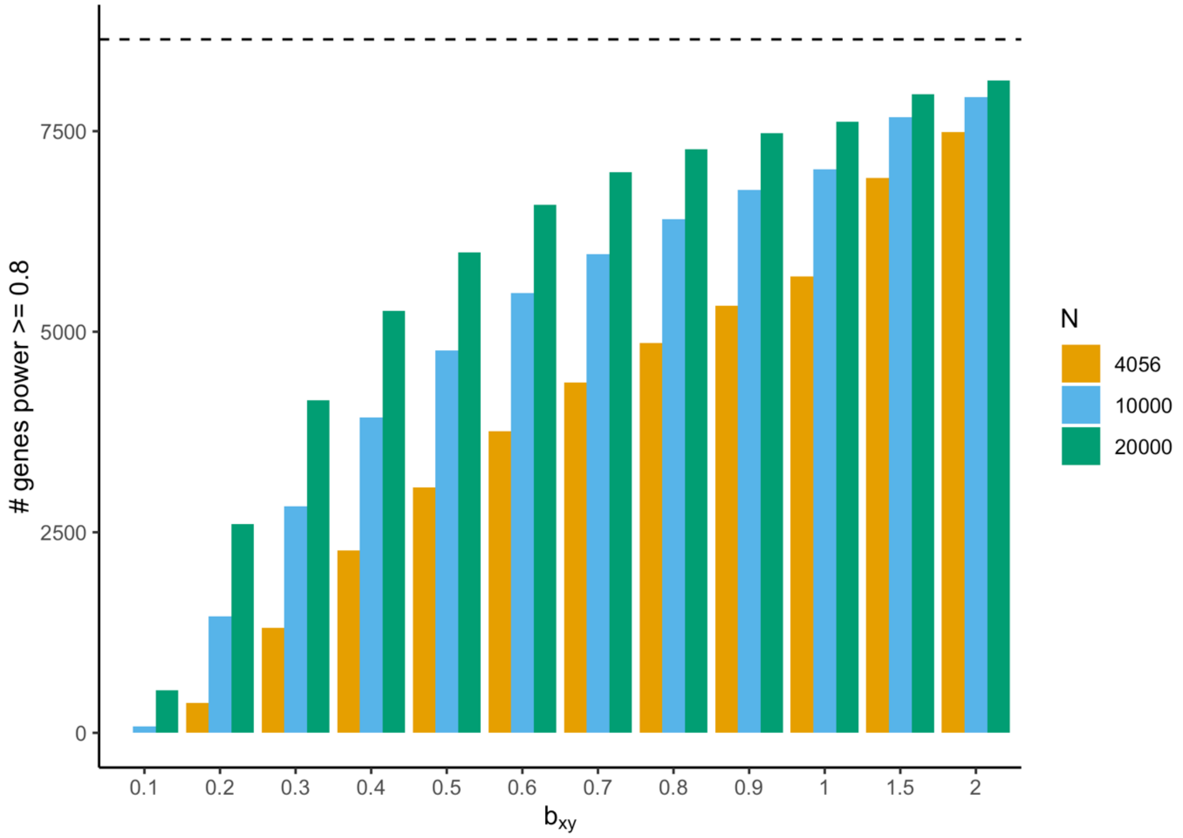

Fig. S6. Increase in statistical power with larger sample sizes.

Number of genes for which we had >0.8 power to detect effects across varying effect sizes in our analysis where we corrected for neighboring GIs (N=4,056) and increasing sample sizes (N=10,000 and N=20,000). b_{xy} (x-axis) represents the effect of gene expression on DNA methylation (1SD change in DNA methylation upon 1SD change in gene expression). b_{xy} is calculated as $b_{xy} = b_{zy}/b_{zx}$, where b_{zy} is the effect of the GI on DNA methylation, and b_{zx} is the effect of the GI on gene expression.

Fig. S7

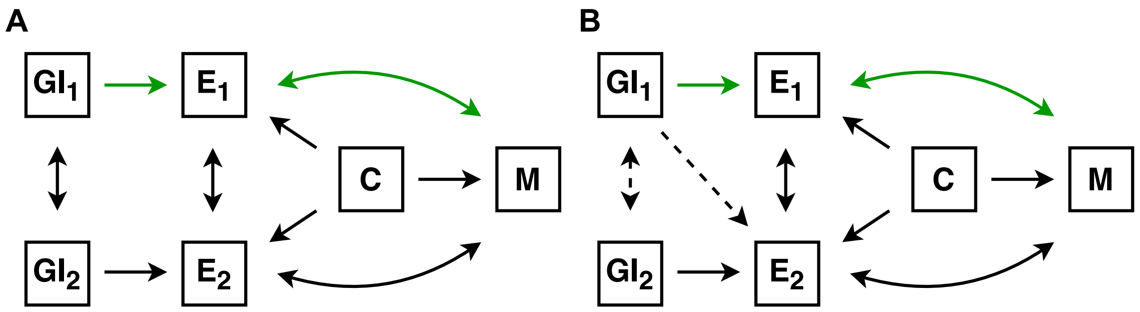

Fig. S7. Diagram showing the presumed relations between genetic instruments, expression and DNA methylation.

Diagrams showing the presumed relations between the genetic instruments (GI), expression (E), confounders (C) and DNA methylation (M). Single arrows indicate a causal effect; double arrows indicate that the causal effect could be in either direction. We aim to identify the effect of a GI (GI_1) on DNA methylation through the expression of its corresponding gene (E_1). Although GIs are not affected by confounding factors, they can be associated with DNA methylation through correlation with a neighboring GI (GI_2) and/or correlation with the expression corresponding to a neighboring GI (E_2) **(a)** Genetic instruments (GIs) can be associated with methylation levels through correlation with nearby GIs. To block this backdoor path, we corrected each GI for all GIs within 1Mb. **(b)** It is possible that a GI is associated with the expression of a nearby gene independent of correlation with the GI corresponding to that gene. We therefore assessed whether significant GIs that shared target CpGs were predictive of each other's gene expression, in which case they were excluded.
