# Supplementary material for: Genome-wide identification of genes regulating DNA methylation using genetic anchors for causal inference
Source: Genome Biol. 2020 Aug 28;21:220. doi: 10.1186/s13059-020-02114-z (PMC7453518; doi:10.1186/s13059-020-02114-z)
Supplement: Supplementary file 19 — Additional file 19. Review history. [file 13059_2020_2114_MOESM19_ESM.docx]

Review History

**First round of review**

**Reviewer 1**

**Are you able to assess all statistics in the manuscript, including the appropriateness of statistical tests used?**

Yes, and I have assessed the statistics in my report.

**Comments to author:**

In this manuscript, Hop et al present a systematic search for (putatively) causal influences of gene expression levels on methylation of various CpGs in human blood. This paper is interesting because it advances a relatively novel type of causal inference method, applied genome-wide, to link genes to a molecular phenotype of interest (here, DNA methylation), thus proposing novel hypotheses about genes that determine DNA methylation. This is substantially different than the more commonly performed methylation QTL analyses (directly linking SNPs to methylation) because here genes are linked (not SNPs) facilitating mechanistic insight.

I think it is, generally speaking, interesting to highlight more of this kind of work, which shows how intermediate phenotypes (esp. gene expression) that are extremely subject to indirect effects and reverse causality can be analyzed by anchoring to genetic variants, thus proposing relationships more likely to be causal.

There are however a couple of points where I see room for improvement in this study, and which might be addressed with additional analyses.

1) the 'genetic instruments' employed explain a low % variance (median R2=4% on the test set) in gene expression levels. This is not in itself unusual, however what needs to be made clear to the reader is that there is a lot of variance in gene expression levels that is not captured by the genetic instruments and therefore the power to find associations with methylation levels via the genetic instruments is not high; many true associations with gene expression will not be recovered, even when a gene is covered by a genetic instrument.
I think we would need to see a power analysis of sorts, to demonstrate how many associations were missed because of this. One wonders why e.g. DNMT1 even though it had a sufficiently predictive genetic instrument, did not record any significant associations.
In particular it is interesting to know how power differs between genes: is the # associations larger in genes having genetic instruments with higher R2s? Can this be used to estimate false negative rates overall in the set of genes considered? Additional analyses would be helpful.

2) the observation that a gene expression level is associated with multiple CpGs is used as evidence to suggest that this gene is more important because it globally controls DNA methylation levels. This makes sense. However one wonders about the following: if global regulators of DNA methylation were sought, why weren't CpGs clustered together (based on methylation patterns) before performing the association tests? (meaning, associations would be sought with the clusters, instead of the individual CpGs)? Power to detect associations would likely be boosted considerably.
Furthermore, what is a bit missing is the analysis of the clustering/co-localization of the multiple CpGs affected by the same gene. Do they co-localize on the genome? Are they enriched in the known binding sites of the TF in question? These are interesting/informative patterns which may help prioritize genes for further follow-up.

3) The function enrichment analyses could be improved. I list several examples (non-exhaustive list) to encourage the authors to increase the rigour in this section of the manuscript.
a- lines 221-223. It would be helpful to see a statistical test (p-value, odds ratio) to show that the proteins exhibiting DNA-binding properties are enriched in the gene set.
b- is there a systematic difference in gene functions between the genes that affect multiple CpGs consistently, and those that affect only 1 CpG (or a low # CpGs)?  This is implied to be the case for TFs however O.R. or p-value does not appear to be supplied. As far as I understand this enrichment is not reported for the other functional categories mentioned (epigenetic factors etc) although it is quite informative in prioritizing genes.
c- can we see a systematic test of GO function enrichments among the non-TF genes that were shown to affect methylation?  (analogous to that in the current lines 190-196)
d- line 205: "the majority of the 36 genes encode proteins that target histone proteins (26 out of 36)" is there a significant enrichment of the histone-targeting proteins in any of the sets? Please specify odds ratios or similar and p-values.
e- concerning the selected examples of non-DNA binding proteins. Were they cherry-picked because an attractive explanation could be speculated about, or are they representatives of broader trends? It would of course be preferred to select examples that are in some way representative. For instance, are the functions (e.g. via GO term analysis or similar) of NFKBIE, NLRC5, SENP7, CDCA7 etc. enriched in the broader set of DNA methylation driving genes? If not, I am not sure they make for the best examples to highlight in the text, and I suggest to the authors to select other genes as examples (those which better represent the functions in the non-TF set of significant genes).

**Reviewer 2**

**Are you able to assess all statistics in the manuscript, including the appropriateness of statistical tests used?**

Yes, and I have assessed the statistics in my report.

**Comments to author:**

Authors take an interesting approach to find genes regulating DNA methylation in two steps: 1) Find genetic instruments which are akin to cis-eQTLs (+/- 100kb) and summarize them into one value, and 2) use these to make correlations with DNA methylation. In doing so, the idea is that the resulting associations between genetic instruments and DNA methylation are "directed" by anchoring dynamic gene expression to static genetic variants. Authors summarize these associations and delves further into specific cis-eQTLs/genetic instruments found within +/-100kb of genes with circumstantial evidence from previous literature. Circumstantial evidence is fine in discussion but not in results.

In essence, this paper represents a slightly modified version of finding mQTLs.  

Specific comments

Line 144-145: Linear regression model equation would be very helpful here. If I understand this correctly, the authors found "directed associations between 2,223 GENETIC INSTRUMENTS and 5,284 CpGs" and NOT "genes". Genetic instruments are derived values comprised of real and imputed components and not biological entities. The authors should try to be as specific as possible when stating their findings. The same confusion applies to TFs and other "proteins". Statements such as "the direction of effects of the TFs on their target CpGs was either skewed towards increased or decreased methylation levels respectively" (line 185) are not clear at all.
Line 165: Authors should report odds ratio for comparison/overlap against each of the previous trans-meQTL studies.
It's interesting that genetic instruments from cis-eQTLs of TFs were associated with DNA methylation in trans, but not cis-eQTLs of Methylation modifiers. It is also interesting that genetic instruments that affect more than 1 CpG, but the question that should be asked that adds validity is NOT how many randomly distributed CpGs, but rather if a cluster of more than one CpG (neighbouring CpGs) can be influenced. This may require DNA modification data with higher resolution or pyrosequencing of specific locus. Mechanistically, it's hard to imagine that a TF will show up in trans to influence only one CpG out of many within X base pairs.
Several grammatical mistakes. Too many to specifically list here.
Line 313-314: There are no findings to suggest that DNA methylation levels were commonly influenced or even influenced by transcription factors.
"... for causal inference" should be removed from the title.

More comments. Most of these comments are on the methods that either 1) helped the authors save more data than they should have or 2) drove stronger statistical significance through transformations.
LD/pleiotropic effects are accounted for during linear regression of GI with DNA methylation. Can LD/haplotypes be corrected for prior to GI generation with lasso regression instead? In other words, GI is expressed as the sum of dosages of each SNP (within 100kb of TSS/TES) multiplied by their effect size, which would mean that SNPs in LD would compound each other.
In contrast to their paper in nature comunications, a lot of missing values for variables (WBCC, age and sex) seem to be imputed for GI generation. Cell count was imputed using pls R package according to van Iterson et al (2017) where it is stated that WBCC is dependent on age and gender some of which were also imputed in the paper's dataset. While I have no issues with imputing data to save data, in this scenario, the order by which imputation was performed may be important.
Outliers in gene expression were not removed, but rather inverse normal transformed, which has become a common practise in some fields without guidance on its usage. For non-normally distributed traits such as gene expression, INT-based tests outperform the standard untransformed association test, both in terms of power and type I error rate control. The authors should justify its usage, and how it drove/influenced "causal" associations with genetic variants and DNA methylation should be addressed.

**Authors Response**

**Point-by-point responses to the reviewers’ comments:**

EBM comments:

In terms of the values of this study, I have general comments as follows.
1) This study predicted a list of “causal” genes, which may regulate the methylation levels of a few CpG sites. However, the regulation specificity of those genes has not been investigated in this study. It is necessary to validate some of them. For example, for transcription factors, it would be pretty straightforward to examine whether their influenced CpGs are located in their putative binding sites.

*We agree with the editor that the identification of enrichment among binding sites of TFs would significantly add to the value of our results. We therefore spent a significant effort on additional analyses to address this question. We analyzed publicly available ChIP-seq data from ENCODE and the Sequence Read Archive (GTRD database [1]) and could indeed confirm enrichment of target CpGs at experimentally determined binding sites. We summarized the transcription factor binding site enrichment for each gene in the figure below (included as figure 3b in the manuscript):*

*Figure 3b. Transcription factor binding site enrichment; each dot represents a transcription factor, with on x-axis the logarithm of the number of target CpGs for that transcription factor (at a gene-level significance level; P < 1.2 x 10-7), and on the y-axis the odds ratio for the enrichment of the target CpGs in its experimentally determined binding sites (ChIP-seq). The size of the dots represents the significance (FDR), and TFs for which the target CpGs were significantly enriched in its binding sites are colored in red.*

*We have included these results in lines 231-237 (Results section):*

*To assess whether TFs may affect DNA methylation directly (i.e. at their binding sites), we leveraged existing ChIP-seq data [1]. For each TF we determined the overlap between the target CpGs (at a gene-level significant threshold: P < 1.2 x 10-7) and its experimentally determined binding sites as compared to a GC-content matched background. ChIP-seq data was available for 59 out of 110 TFs affecting multiple CpGs (at P < 1.2 x 10-7). For one third of these TFs (N = 20), target CpGs were significantly enriched for co-localization with their respective TF binding sites (FDR < 0.05; figure 3b, table S12).*

*And lines 820-835 (Methods section):*

*We obtained transcription factor ChIP-seq peaks called with the MACS2 software from the GTRD database (*<http://gtrd.biouml.org/>*), which contains uniformly processed ChIP-seq data from ENCODE and the Sequence Read Archive (SRA) [1–4]. For 59 out of the 110 identified transcription factors associated with multiple CpGs (2 or more), at least 1 ChIP-seq experiment was available. For each TF we overlapped target CpG locations (at a gene-level significant threshold, P < 1.2 x 10-7) and its experimentally determined binding sites (ChIP-seq peaks). If multiple experiments were available for a specific TF, we determined the overlap per experiment. The HOMER software was used to generate a background set for each TF with the same GC-content distribution as the target CpGs (100,000 regions) [5]. We performed a Fisher’s exact test to determine whether the target CpGs overlapped with ChIP-seq peaks more often than the background regions. We employed a two-step approach to account for multiple testing, where first the Simes procedure was used to control for multiple experiments available per TF (since they are expected to be correlated), and second the Benjamin-Hochberg procedure was used to control the FDR among the tested TFs [6].*

2) The authors claimed that the results of this study can guide follow-up studies for understanding epigenetic regulation, but they did not provide an example to show the value of their prediction. I guess this study may be interesting to population genetics filed, but the values of the predicted results are limited to epigenetic community.

*Thank you for priming us to more explicitly discuss the added value of our results. We took the opportunity to substantially extend our manuscript with detailed analyses of transcription factor binding sites (as discussed in the previous point), exploring overlap with disease-causing genes, and intersecting our data with results of previous epigenome-wide association studies. We believe this significantly increased the interest of our paper to the wider audience of Genome Biology. Below is a new paragraph that we now include in the Discussion summarizing our effort (lines 502-526):*

*We envision multiple applications of our findings. First, we identified many genes that were previously unknown to be involved in the regulation of DNA methylation. Importantly, the genes were enriched for transcription factors that, in turn, commonly affected DNA methylation at their binding sites, thereby providing new targets for studies into epigenomic regulation. Second, epigenetic dysregulation is a hallmark of many diseases and, in line with this, mutations in genes regulating the epigenome are increasingly reported to be involved in Mendelian disease [7]. We found that 200 out of the 818 genes we implicated in the regulation of DNA methylation were known disease genes (OMIM; table S18) [8]. Our results may aid in elucidating downstream effects of these disease genes. An interesting example in this regard is CDCA7L, which we found to affect DNA methylation throughout the genome in a similar fashion as CDCA7. Mutations in CDCA7 lead to the ICF syndrome, a syndrome characterized by hypomethylation in pericentric repeats [9]. Since we found that CDCA7L has similar effects on DNA methylation, it may be hypothesized that mutations in CDAC7L lead to similar phenotypes. Finally, altered DNA methylation patterns have been reported for many environmental exposures and traits using epigenome-wide association studies (EWAS). However it often remains unclear how these patterns are established [10]. The target CpGs identified in our analyses can aid in interpreting EWAS results and may point to the signal transduction pathways relaying external and internal stimuli to the methylome. To illustrate this point, we overlapped the identified target CpGs with existing EWAS results (table S13) and found that target CpGs of several genes were enriched for trait-associated CpGs, including Werner Syndrome (SENP7, a SUMO peptidase; SUMO modifies the Werner Syndrome gene product [11,12]), auto-immune diseases and inflammatory markers (NLRC5, a key regulator of MHC class I-dependent immune response [13]), and obesity/BMI (NFKBIE and NFKB1; NFκB is a central regulator of inflammatory response, including metabolism-related inflammation[14]).*

Reviewer reports:

Reviewer #1

In this manuscript, Hop et al present a systematic search for (putatively) causal influences of gene expression levels on methylation of various CpGs in human blood. This paper is interesting because it advances a relatively novel type of causal inference method, applied genome-wide, to link genes to a molecular phenotype of interest (here, DNA methylation), thus proposing novel hypotheses about genes that determine DNA methylation. This is substantially different than the more commonly performed methylation QTL analyses (directly linking SNPs to methylation) because here genes are linked (not SNPs) facilitating mechanistic insight.

I think it is, generally speaking, interesting to highlight more of this kind of work, which shows how intermediate phenotypes (esp. gene expression) that are extremely subject to indirect effects and reverse causality can be analyzed by anchoring to genetic variants, thus proposing relationships more likely to be causal.

*We very much appreciate the reviewer’s positive assessment of our work.*

There are however a couple of points where I see room for improvement in this study, and which might be addressed with additional analyses.

1) the 'genetic instruments' employed explain a low % variance (median R2=4% on the test set) in gene expression levels. This is not in itself unusual, however what needs to be made clear to the reader is that there is a lot of variance in gene expression levels that is not captured by the genetic instruments and therefore the power to find associations with methylation levels via the genetic instruments is not high; many true associations with gene expression will not be recovered, even when a gene is covered by a genetic instrument.

I think we would need to see a power analysis of sorts, to demonstrate how many associations were missed because of this. One wonders why e.g. DNMT1 even though it had a sufficiently predictive genetic instrument, did not record any significant associations.

In particular it is interesting to know how power differs between genes: is the # associations larger in genes having genetic instruments with higher R2s? Can this be used to estimate false negative rates overall in the set of genes considered? Additional analyses would be helpful.

*In compliance with the reviewer’s comments, we have performed power analyses using an established method [15] and made a series of changes throughout our manuscript to incorporate the results and implications (A-G below).*

*A. As indicated by the reviewer, the power to detect associations is strongly dependent on the proportion of variance in expression explained by a genetic instrument (left figure below). On the right figure below (included as supplemental figure 2) we present the number of genes for which we had >0.8 power to detect varying effect sizes (x-axis; bxy), showing the difference in power between genes (we included the power for each individual gene in table S1). As expected, correction for neighboring GIs increases specificity, but does so at the cost of reducing power to detect associations as can be seen on the figure on the right below (the reduction in power is dependent on the correlation between the genetic instrument in question and the nearby genetic instruments). We already mentioned this point in the discussion but now support this point with the power calculation.*

*­­­­*

*We describe the findings in lines 177-189 (Results section):*

*We performed post-hoc power analyses to assess the power we had to detect varying effect sizes for each gene tested (figure S2, table S1) [15]. In the uncorrected analysis (not corrected for neighboring GIs), we had >0.8 power to detect effect sizes of 1 SD (1 standard deviation change in DNA methylation upon 1 standard deviation change in expression) for about 85% of the tested genes, and for about 50% of the genes (4,475) we had >0.8 power to detect effect sizes of 0.5 SD (figure S2). Correcting for neighboring GIs is required to identify specific genes (instead of genomic regions with multiple correlated genes), but does so at the cost of reduced power. Correction left 5,685 genes (compared to 7,299) with power >0.8 to detect effect sizes of 1 SD, and left 3,061 genes (compared to 4,475) with >0.8 power to detect effect sizes of 0.5 SD (figure S2). This analysis shows that for the majority of tested genes we were well-powered to detect large effects and for over a third of the genes we were well-powered to detect medium effect sizes. We included the explained variance and power across varying effect sizes for each gene in table S1.*

*and in lines 801-807 (methods section):*

*We calculated statistical power to detect associations at a two-sided α of 1.4 x 10-11 based on the proportion of variance in gene expression explained by the genetic instruments, the sample size, and varying hypothetical effect sizes [15]. We evaluated the statistical power for both the uncorrected analysis (not including nearby genetic instruments) and the corrected analysis (including nearby genetic instruments, <1Mb). For the corrected analysis we calculated power using the proportion of variance in gene expression explained taking into account the neighboring GIs (partial R2).*

*B. In addition, we examined the increase in power for higher sample sizes and note that especially for smaller effect sizes, the number of identified genes and target CpGs can be significantly expanded by applying our method larger datasets (this figure is included as supplemental figure 6 and mentioned in the discussion; lines 496-501):*

*C. In table S1, we now included the power for each gene across varying effect sizes. Using this table, researchers can assess whether specific genes of interest were missed because of limited power.*

*D. Based on the insights from the power analyses, we updated our assessment of DNMT3A and DNMT1 (lines 253-261 in the Results section). We note that the R2 values that we mentioned in the original manuscript (R2 DNMT3A = 0.05, R2 DNMT1 = 0.04) represent the uncorrected explained variance (i.e. not corrected for neighboring GIs). Although for DNMT3A the corrected R2 is quite similar (0.049 compared to 0.050), for DNMT1 the corrected R2 is significantly lower than the uncorrected R2 (0.0009 compared to 0.04). This could explain why we did identify significant associations for DNMT3A, but did not identify any significant associations for DNMT1. We do note however, that also in the uncorrected analyses (where power for DNMT1 and DNMT3A was very similar), we did not find any significant associations for DNMT1a, as can be seen in the figure below (A = uncorrected test-statistics DNMT3A; B = corrected test-statistics DNMT3A; C = uncorrected test-statistics DNMT1; D = corrected test-statistics DNMT1). So, our original interpretation of the different results for DNMT3A and DNMT1 remains unchanged.*

*E. As indicated by the reviewer, we also assessed whether the number of associations is larger in genes having genetic instruments with higher R2 values. The explained variance of genes associated with multiple target CpGs was on average 3% higher (median change = 1.1%) than in genes for which we* *found only 1 significant association:*

*We now mention in the discussion (lines 493-494) that for significant genes, limited power may have resulted in the identification of a portion of the true number of CpG sites affected by the respective gene.*

*F. Moreover, we realized the enrichment of TFs among the identified genes may be influenced by the variance explained in the genetic instruments (i.e. if genetic instruments for TFs generally explain more of the variation in expression, this could explain why we found more TFs than non-TFs). We found however that both in the uncorrected (for neighboring GIs) and corrected analysis the explained variance was in fact higher in non-TFs than TFs (p = 6.72 x 10-6 for the uncorrected analysis and p = 6.32* *x 10-8 for the corrected analysis; included in lines 205-206 and supplemental figure 3):*

*G. Finally, we note that we referred to our results as a ‘resource’ in the original manuscript. Upon reevaluation, we think that this term suggests that we provide an exhaustive list of genes (i.e. a database) of genes involved in the regulation of DNA methylation. Given that 1) we could not test all genes, since we did not have a sufficient predictive genetic instrument for each gene and 2) the power to detect associations varies across genes, we think that ‘resource’ is not an optimal description for our results, and therefore we removed the term from the manuscript.*

2.1) the observation that a gene expression level is associated with multiple CpGs is used as evidence to suggest that this gene is more important because it globally controls DNA methylation levels. This makes sense. However one wonders about the following: if global regulators of DNA methylation were sought, why weren't CpGs clustered together (based on methylation patterns) before performing the association tests? (meaning, associations would be sought with the clusters, instead of the individual CpGs)? Power to detect associations would likely be boosted considerably.

Furthermore, what is a bit missing is the analysis of the clustering/co-localization of the multiple CpGs affected by the same gene. Do they co-localize on the genome?

*To accommodate the reviewer’s question, we added the following information to the manuscript:*

*We tested whether target CpGs were co-localized (<1kb) and found that target CpGs were indeed co-localized on the genome: for genes affecting DNA methylation at more than 1 CpG-site, on average 33% of the target CpGs were colocalized (<1kb) with at least one other target CpG (lines 166-169). In total, the 2,384 unique target CpGs were located in 1,915 distinct regions (consecutive probes within <1kb).*

*Moreover, CpGs affected by TFs more often co-localized than CpGs affected by non-TFs (lines 211-214). For TFs affecting more than 1 CpG, on average 45% of the target CpGs were colocalized with at least one other target CpG (<1kb), which was a significant enrichment compared to non-TFs (average non-TFs=25%, OR = 2.5, p = 2.2 x 10-21).*

*In supplementary table 5, we included the number of independent regions affected by each TF (i.e. clusters of CpGs within <1kb of each other), and also the number of clusters of a particular size (e.g. number of associated clusters that contain 2 CpGs, 3 CpGs, etc.).*

2.2) Are they enriched in the known binding sites of the TF in question? These are interesting/informative patterns which may help prioritize genes for further follow-up.

*We agree that the identification of enrichment among binding sites of TFs would significantly add to the value of our results. We therefore spent a significant effort on additional analyses to address this question. We analyzed publicly available ChIP-seq data from ENCODE and the Sequence Read Archive (GTRD database [1]) and could indeed confirm enrichment of target CpGs at experimentally determined binding sites. We summarized the transcription factor binding site enrichment for each gene in the figure below (included as figure 3b in the manuscript):*

*Figure 3b. Transcription factor binding site enrichment; each dot represents a transcription factor, with on x-axis the logarithm of the number of target CpGs for that transcription factor (at a gene-level significance level; P < 1.2 x 10-7), and on the y-axis the odds ratio for the enrichment of the target CpGs in its experimentally determined binding sites (ChIP-seq). The size of the dots represents the significance (FDR), and TFs for which the target CpGs were significantly enriched in its binding sites are colored in red.*

*We have included these results in lines 231-237 (Results section):*

*To assess whether TFs may affect DNA methylation directly (i.e. at their binding sites), we leveraged existing ChIP-seq data [1]. For each TF we determined the overlap between the target CpGs (at a gene-level significant threshold: P < 1.2 x 10-7) and its experimentally determined binding sites as compared to a GC-content matched background. ChIP-seq data was available for 59 out of 110 TFs affecting multiple CpGs (at P < 1.2 x 10-7). For one third of these TFs (N = 20), target CpGs were significantly enriched for co-localization with their respective TF binding sites (FDR < 0.05; figure 3b, table S12).*

*And lines 820-835 (Methods section):*

*We obtained transcription factor ChIP-seq peaks called with the MACS2 software from the GTRD database (*<http://gtrd.biouml.org/>*), which contains uniformly processed ChIP-seq data from ENCODE and the Sequence Read Archive (SRA) [1–4]. For 59 out of the 110 identified transcription factors associated with multiple CpGs (2 or more), at least 1 ChIP-seq experiment was available. For each TF we overlapped target CpG locations (at a gene-level significant threshold, P < 1.2 x 10-7) and its experimentally determined binding sites (ChIP-seq peaks). If multiple experiments were available for a specific TF, we determined the overlap per experiment. The HOMER software was used to generate a background set for each TF with the same GC-content distribution as the target CpGs (100,000 regions) [5]. We performed a Fisher’s exact test to determine whether the target CpGs overlapped with ChIP-seq peaks more often than the background regions. We employed a two-step approach to account for multiple testing, where first the Simes procedure was used to control for multiple experiments available per TF (since they are expected to be correlated), and second the Benjamin-Hochberg procedure was used to control the FDR among the tested TFs [6].*

3) The function enrichment analyses could be improved. I list several examples (non-exhaustive list) to encourage the authors to increase the rigour in this section of the manuscript.

a- lines 221-223. It would be helpful to see a statistical test (p-value, odds ratio) to show that the proteins exhibiting DNA-binding properties are enriched in the gene set.

*We have included the odds ratio and p-value (fisher’s exact test) for the number of DNA-binding proteins among the ‘other’ genes (OR = 0.91, P > 0.05) (Lines 266-267). Thus, there is no significant enrichment of DNA-binding proteins among the ‘other’ genes (that is: genes not annotated as TFs or Epifactor).*

b- is there a systematic difference in gene functions between the genes that affect multiple CpGs consistently, and those that affect only 1 CpG (or a low # CpGs)?  This is implied to be the case for TFs however O.R. or p-value does not appear to be supplied.

As far as I understand this enrichment is not reported for the other functional categories mentioned (epigenetic factors etc) although it is quite informative in prioritizing genes.

*We thank the reviewer for pointing this out. Although we did show that the TF enrichment becomes increasingly stronger when focusing on genes with increasing number of target CpGs (figure 3a), we did indeed not formally test whether genes with multiple target CpGs were enriched for transcription factors. We have therefore included the odds-ratio and p-value (fisher’s exact test) for the number of TFs affecting >1 CpG compared to the non-TFs affecting >1 CpG (OR = 3.45, P = 3.1 x 10-10) (lines 209-211). Similarly, we have stated the number of EpiFactors for which we identified more than 1 CpG (12 genes; OR = 0.82, P > 0.05; lines 244-246).*

c- can we see a systematic test of GO function enrichments among the non-TF genes that were shown to affect methylation?  (analogous to that in the current lines 190-196)

*We performed a GO enrichment analysis (DAVID) in the non-TF genes. This did not yield significant enrichments; we discuss this in more detail in point 3e.*

d- line 205: "the majority of the 36 genes encode proteins that target histone proteins (26 out of 36)" is there a significant enrichment of the histone-targeting proteins in any of the sets? Please specify odds ratios or similar and p-values.

*We included the odds ratio and p-value (fisher’s exact test) for the number of histone-targeting proteins among the EpiFactors (OR = 1.13; P > 0.05; lines 246-247). We do think that the identification of histone-modifying proteins is interesting to mention (although it does not represent an enrichment within the significant EpiFactors). For example, the significant EpiFactors include IKZF1 (known to associate with components of the histone deacetylase complex) which we found to be associated with DNA methylation at its binding sites. Given the increasing interest in the interplay between histone modifications and DNA methylation [16], we think these results may provide valuable leads for follow-up.*

e- concerning the selected examples of non-DNA binding proteins. Were they cherry-picked because an attractive explanation could be speculated about, or are they representatives of broader trends? It would of course be preferred to select examples that are in some way representative. For instance, are the functions (e.g. via GO term analysis or similar) of NFKBIE, NLRC5, SENP7, CDCA7 etc. enriched in the broader set of DNA methylation driving genes? If not, I am not sure they make for the best examples to highlight in the text, and I suggest to the authors to select other genes as examples (those which better represent the functions in the non-TF set of significant genes).

*We performed a GO enrichment analysis among the non-DNA binding proteins, but did not find significant enrichments, presumably because the function of this group of genes is heterogeneous. We now make clear that the selected examples represent the top 5 non-DNA binding genes in terms of number of target CpGs (we now explicitly state this in the text, lines 271-275). We agree with the reviewer that these genes do not necessarily represent a broader trend among the non-DNA-binding genes. The examples provide insights into several plausible biological mechanisms through which DNA methylation can be affected by proteins that do not directly bind to DNA (i.e. post-translational regulation, post-translational modification and attraction of histone-modifying proteins).*

Reviewer #2

Authors take an interesting approach to find genes regulating DNA methylation in two steps: 1) Find genetic instruments which are akin to cis-eQTLs (+/- 100kb) and summarize them into one value, and 2) use these to make correlations with DNA methylation. In doing so, the idea is that the resulting associations between genetic instruments and DNA methylation are "directed" by anchoring dynamic gene expression to static genetic variants.

Authors summarize these associations and delves further into specific cis-eQTLs/genetic instruments found within +/-100kb of genes with circumstantial evidence from previous literature. Circumstantial evidence is fine in discussion but not in results.

*The presented examples were included as interesting case studies exploring some of the possible biological functions underlying non-DNA-binding genes that affect DNA methylation. To this end we chose the top 5 non-DNA-binding genes in terms of the number of target CpGs affected. To prevent the suggestion that these examples prove the validity of all results or do represent a broader trend among the non-DNA-binding genes, we rephrased lines 271-275 in the results section:*

*GO term enrichment analysis did not reveal significant functions underlying these genes. To explore possible biological functions among these genes, we provide case studies below for the five genes from this set that were associated with the most target CpGs: SENP7 (189 target CpGs), CDCA7 (79 target CpGs), NFKBIE (76 target CpGs), CDCA7L (47 target CpGs) and NLRC5 (43 target CpGs).*

*And in the discussion section (lines 420-428):*

*Second, exploration of the top five non-DNA-binding genes with the highest number of associated target CpGs, suggests that protein-protein interactions are among the possible mechanisms. The mechanisms include post-translational regulation (NFKBIE encodes for IκBε which retains NF-kB in the cytoplasm [17]), post-translational modification (SENP7: deSUMOylates the repressor KAP1 [18]), and recruitment of epigenetic proteins to specific target sites through association with a DNA-binding protein (NLRC5 associates with a protein complex in MHC-I region [13]).*

*Second, we note that including case studies/examples in the results section is in itself not unusual, three out of the four mQTL studies that we cited in our manuscript also present examples in their results [19–21].*

In essence, this paper represents a slightly modified version of finding mQTLs.

*We believe that our approach differs from traditional QTL analysis in key aspects:*

*Our analysis does not result in single genetic variants that are associated with DNA methylation, but genes, which inherently have a clearer biological interpretation.*

*Although some mQTL-studies have studied trans-meQTL effects in relation to cis expression, these analyses do not control for LD/pleiotropy among neighboring genes, and as we showed here and previously [18], this hampers the ability to detect the specific gene through which DNA methylation is affected due to horizontal pleiotropy as induced by linkage disequilibrium.*

*Using multi-SNP predictors of gene expression significantly increases power to detect effects as compared with using single variants [18,22,23]. Power is further enhanced because multiple testing is reduced (testing 10-20 thousand genes instead of millions of genetic variants) [21,22].*

Specific comments

Line 144-145: Linear regression model equation would be very helpful here.

*We included linear regression model equations in lines 774-780 and lines 783-788. We also included a mathematical notation for the construction of the genetic instruments in lines 757-759.*

If I understand this correctly, the authors found "directed associations between 2,223 GENETIC INSTRUMENTS and 5,284 CpGs" and NOT "genes". Genetic instruments are derived values comprised of real and imputed components and not biological entities.

*Genetic instruments are widely applied to identify directed associations between two variables, most notably in Mendelian Randomization or, more recently, so called TWAS approaches using imputed gene expression [22–24]. In these cases, it is common to use the term for the variable that is instrumented, like in our case ‘gene’. We agree with the reviewer, that it should be well-defined how the genetic instrument is constructed and which assumptions and limitations apply to the analysis. In more detail:*

*Usage of the term ‘gene’ when referring to the genetic component of gene expression, is a conventional term and widely used in literature [22,23,25].*

*The fundamental assumption in our method is that that the causal path is as follows:*

$$GI\to gene expression\to DNA methylation$$

*Although the genetic instruments explain only a fraction of the actual variation in gene expression, one would expect that the non-genetic component of variation in gene expression has the same downstream effects as the genetic component of variation in expression, and therefore the genetic instrument act as a valid proxy for the gene. Of course, these cis genetic variants may be pleiotropic, which would invalidate the main assumption. We therefore systematically considered LD/pleiotropy among neighboring genes, although we acknowledge that it is still possible that a genetic instrument may have pleiotropic effects through other mechanisms than expression such as interchromosomal contacts (lines 478-484 in the Discussion section). So, the identification of directed associations between genes and trans DNA methylation is valid under the condition that the above-mentioned assumptions hold. In line with previous literature, we believe it is justified to use the word ‘gene’, because we clearly convey to the reader which assumptions are made. We agree with the reviewer that being transparent about the assumptions and limitations is key. Therefore, we rephrased the limitation section of the discussion to again state the assumption inherent to the method explicitly (lines 478-484):*

*Second, the main assumption in our analysis is that the genetic instruments affect DNA methylation through their effect on gene expression. Although we systematically considered LD/pleiotropy among neighboring genes, the genetic instruments may have pleiotropic effects on unmeasured genes. In addition, although trained to capture variation in gene expression, genetic instruments may inadvertently be associated with trans-DNA methylation through other mechanisms than expression such as interchromosomal contacts [21].*

The authors should try to be as specific as possible when stating their findings. The same confusion applies to TFs and other "proteins". Statements such as "the direction of effects of the TFs on their target CpGs was either skewed towards increased or decreased methylation levels respectively" (line 185) are not clear at all.

*To convey our results more clearly, we changed several sentences:*

*Lines 193-196 (Results section):*

*We observed that these genes, often consistently, either increased or decreased DNA methylation at their target CpGs (Figure 2a).*

*Lines 214-218 (Results section):*

*The majority of TFs either consistently increased or consistently decreased DNA methylation at their target CpGs: a significant skew in the direction of effect was present for 20 out of 23 TFs that were associated with at least 10 CpGs (6 consistently decreased, and 14 consistently increased DNA methylation at the target CpGs respectively).*

*And lines 385-388 (Discussion section):*

*Our results highlight a role of TFs. TFs were overrepresented among the identified genes and either consistently increased or decreased DNA methylation at their target CpGs.*

*In addition, we consulted a native speaker who read the manuscript in detail, which resulted in the correction of grammatical mistakes and improved readability throughout the manuscript.*

Line 165: Authors should report odds ratio for comparison/overlap against each of the previous trans-meQTL studies.

*We have included the odds ratio for the overlap between trans-meQTL probes identified in previous studies in the manuscript (OR = 103; P < 1 x 10-32). In Table S6 (included below) we included the enrichment for the overlap between the probes identified in this study and each individual trans-meQTL study. Note, the crucial difference between our study and previous QTL studies is that our systematic analysis using genetic instruments directly points to the genes involved, while for a regular QTL analysis it remains unclear through which gene a genetic variant operates (and relies on post-hoc interpretation which often is based on oversimplified assumptions (like nearest gene) and cannot account for pleiotropy).*

| *Study* | *Number of overlapping CpGs* | *Number of overlapping CpGs with corresponding GI and trans-meQTL SNP in close proximity (<1Mb)* | *Enrichment* |
| --- | --- | --- | --- |
| *Lemire et al. (2015)* | *504* | *489* | *OR=97; P < 1 x 10-32* |
| *Gaunt et al. (2016)* | *413* | *408* | *OR=116; P < 1 x 10-32* |
| *Huan et al. (2019)* | *1,520* | *1,482* | *OR=88; P < 1 x 10-32* |
| *Total* | *1,628* | *1,589* | *OR=103; P < 1 x 10-32* |

It’s interesting that genetic instruments from cis-eQTLs of TFs were associated with DNA methylation in trans, but not cis-eQTLs of Methylation modifiers. It is also interesting that genetic instruments that affect more than 1 CpG, but the question that should be asked that adds validity is NOT how many randomly distributed CpGs, but rather if a cluster of more than one CpG (neighbouring CpGs) can be influenced. This may require DNA modification data with higher resolution or pyrosequencing of specific locus. Mechanistically, it's hard to imagine that a TF will show up in trans to influence only one CpG out of many within X base pairs.

*We agree that evaluating clustering of CpGs is of interest.*

*We tested whether target CpGs were co-localized (<1kb) and found that target CpGs were indeed co-localized on the genome: for genes affecting DNA methylation at more than 1 CpG-site, on average 33% of the target CpGs were colocalized (<1kb) with at least one other target CpG (lines 166-169). In total, the 2,384 unique target CpGs were located in 1,915 distinct regions (consecutive probes within <1kb).*

*Moreover, CpGs affected by TFs more often co-localized than CpGs affected by non-TFs (lines 211-214). For TFs affecting more than 1 CpG, on average 45% of the target CpGs were colocalized with at least one other target CpG (<1kb), which was a significant enrichment compared to non-TFs (average non-TFs=25%, OR = 2.5, p = 2.2 x 10-21).*

*In supplementary table 5, we included the number of independent regions affected by each TF (i.e. clusters of CpGs within <1kb of each other), and also the number of clusters of a particular size (e.g. number of associated clusters that contain 2 CpGs, 3 CpGs, etc.).*

*We agree with the reviewer that DNA methylation data with higher resolution, such as bisulfite sequencing, would be a valuable next step and mention it in the discussion (lines 494-501):*

*An additional factor reducing power is the correction for nearby GIs, which is required to obtain specific associations but at the same time leads to the loss of true effects. Hence, we expect that the genes affecting distant DNA methylation we report on here can be significantly expanded by applying our method to datasets obtained using more comprehensive DNA methylation profiling assays than the 450k array, to larger sample numbers (see power analyses in figure S6), and, in particular, to other tissues than blood.*

Several grammatical mistakes. Too many to specifically list here.

*We thank the reviewer for pointing this out. We consulted a native speaker who read the manuscript in detail. This led to the correction of several grammatical mistakes and improved the readability of several sentences (changes are tracked in the revised manuscript).*

Line 313-314: There are no findings to suggest that DNA methylation levels were commonly influenced or even influenced by transcription factors.

*To address the criticism of the reviewer, we did four things:*

*We removed the word ‘commonly’ in the manuscript, and instead we now explicitly state what we found, namely: ‘the identified genes were enriched for transcription factors’.*

*Expanded on limitations and careful interpretation of findings, in particular that our method does not distinguish between genes that directly affect DNA methylation (e.g. binding of a transcription factor that leads to a loss/increase of DNA methylation at its binding sites) and genes that indirectly affect DNA methylation, hence are on the causal path but not themselves mechanistically (e.g. a TF that regulates the expression of a DNA methylation modifier.) This is included in lines 469-478 (Discussion section):*

*We should, however, also note limitations of our method. First, our method does not distinguish between mechanistically direct or indirect effects of gene expression on DNA methylation. An example in this regard is NFKBIE, which affects DNA methylation through inhibition of the transcription factor NFκB. Similarly, TFs could affect DNA methylation indirectly through the regulation of another gene. We note, however, that CpGs affected by TFs commonly co-localized with their respective binding sites, favoring the interpretation of a direct effect.*

*To further accommodate the reviewer’s concern, we replaced the term ‘influence’ with ‘affect’ throughout the manuscript, in accordance with our published work on the identification of directed associations between genes [18]. We think that the less specific term ‘affect’ fits better, since ‘influence’ may be more readily be interpreted as a direct mechanistic effect.*

*We performed transcription factor binding site enrichment analyses, showing that the target CpGs of a substantial number of TFs were enriched among their respective binding sites. Thus, we found evidence for a direct effect for several TFs.*

*We have included these results in lines 231-237 (Results section):*

*To assess whether TFs may affect DNA methylation directly (i.e. at their binding sites), we leveraged existing ChIP-seq data [1]. For each TF we determined the overlap between the target CpGs (at a gene-level significant threshold: P < 1.2 x 10-7) and its experimentally determined binding sites as compared to a GC-content matched background. ChIP-seq data was available for 59 out of 110 TFs affecting multiple CpGs (at P < 1.2 x 10-7). For one third of these TFs (N = 20), target CpGs were significantly enriched for co-localization with their respective TF binding sites (FDR < 0.05; figure 3b, table S12).*

*And lines 820-835 (Methods section):*

*We obtained transcription factor ChIP-seq peaks called with the MACS2 software from the GTRD database (*<http://gtrd.biouml.org/>*), which contains uniformly processed ChIP-seq data from ENCODE and the Sequence Read Archive (SRA) [1–4]. For 59 out of the 110 identified transcription factors associated with multiple CpGs (2 or more), at least 1 ChIP-seq experiment was available. For each TF we overlapped target CpG locations (at a gene-level significant threshold, P < 1.2 x 10-7) and its experimentally determined binding sites (ChIP-seq peaks). If multiple experiments were available for a specific TF, we determined the overlap per experiment. The HOMER software was used to generate a background set for each TF with the same GC-content distribution as the target CpGs (100,000 regions) [5]. We performed a Fisher’s exact test to determine whether the target CpGs overlapped with ChIP-seq peaks more often than the background regions. We employed a two-step approach to account for multiple testing, where first the Simes procedure was used to control for multiple experiments available per TF (since they are expected to be correlated), and second the Benjamin-Hochberg procedure was used to control the FDR among the tested TFs [6].*

"... for causal inference" should be removed from the title.

*The term ‘for causal inference’ is a common way to refer to the class of methods we employ (for example see [26]) and is used to signal the kind of method we employed to the reader.*

More comments. Most of these comments are on the methods that either 1) helped the authors save more data than they should have or 2) drove stronger statistical significance through transformations.

LD/pleiotropic effects are accounted for during linear regression of GI with DNA methylation. Can LD/haplotypes be corrected for prior to GI generation with lasso regression instead? In other words, GI is expressed as the sum of dosages of each SNP (within 100kb of TSS/TES) multiplied by their effect size, which would mean that SNPs in LD would compound each other.

*We appreciate it that the reviewer pointed out this unclarity. We used LASSO regression to generate the GIs, which accounts for correlation (LD) among the genetic variants, and therefore the SNPs in the GIs should not compound each other. However, although this accounts for correlation among variants included within each genetic instrument, this does not account for correlation between genetic instruments. For this reason, we adjusted for neighboring GIs when testing for an association between genetic instrument and trans DNA methylation levels.*

*To convey more clearly that we used LASSO regression for variable selection, we rephrased lines 132-134 in the Results section:*

*In the training set (1/3 of the data, 1,119 individuals), we obtained a GI for the expression of each gene, which consisted of 1 or more SNPs selected by applying LASSO regression to nearby genetic variants [27].*

In contrast to their paper in nature comunications, a lot of missing values for variables (WBCC, age and sex) seem to be imputed for GI generation. Cell count was imputed using pls R package according to van Iterson et al (2017) where it is stated that WBCC is dependent on age and gender some of which were also imputed in the paper's dataset. While I have no issues with imputing data to save data, in this scenario, the order by which imputation was performed may be important.

*For the imputation of WBCC, we included non-imputed age and sex as covariates (i.e. only using data for which reported age and sex were available), and prediction models for age and sex were trained using univariate models. Thus, they do not rely on imputed data, which would indeed have been suboptimal. We now clarify in the Methods section (lines 719-721):*

*Missing observations in the measured white blood cell counts (WBCC) were imputed using the R package pls, adjusting for reported age and sex, as described earlier [28].*

Outliers in gene expression were not removed, but rather inverse normal transformed, which has become a common practise in some fields without guidance on its usage. For non-normally distributed traits such as gene expression, INT-based tests outperform the standard untransformed association test, both in terms of power and type I error rate control. The authors should justify its usage, and how it drove/influenced "causal" associations with genetic variants and DNA methylation should be addressed.

*We included three references justifying the use of INT (lines 664-666 in the Methods section), which indeed show that INT-based tests result in type I error rate control, although it may result in some loss of power [29–31].*

*In addition, we assessed whether any of the significant associations were induced by the INT-transformation by rerunning the association tests for the significant associations using non-transformed data. We found that test-statistics using either non-INT or INT-transformed data were very similar and thus using INT-transformation did not drive stronger statistical associations:*

*References*

*1. Yevshin I, Sharipov R, Kolmykov S, Kondrakhin Y, Kolpakov F. GTRD: a database on gene transcription regulation—2019 update. Nucleic Acids Res. 2019;47:D100–5.*

*2. Zhang Y, Liu T, Meyer CA, Eeckhoute J, Johnson DS, Bernstein BE, et al. Model-based Analysis of ChIP-Seq (MACS). Genome Biol. 2008;9:R137.*

*3. Landt SG, Marinov GK, Kundaje A, Kheradpour P, Pauli F, Batzoglou S, et al. ChIP-seq guidelines and practices of the ENCODE and modENCODE consortia. Genome Res. 2012;22:1813–31.*

*4. Kodama Y, Shumway M, Leinonen R, on behalf of the International Nucleotide Sequence Database Collaboration. The sequence read archive: explosive growth of sequencing data. Nucleic Acids Res. 2012;40:D54–6.*

*5. Heinz S, Benner C, Spann N, Bertolino E, Lin YC, Laslo P, et al. Simple Combinations of Lineage-Determining Transcription Factors Prime cis-Regulatory Elements Required for Macrophage and B Cell Identities. Mol Cell. 2010;38:576–89.*

*6. Luijk R, Goeman JJ, Slagboom EP, Heijmans BT, van Zwet EW. An alternative approach to multiple testing for methylation QTL mapping reduces the proportion of falsely identified CpGs. Bioinformatics. 2015;31:340–5.*

*7. Bjornsson HT. The Mendelian disorders of the epigenetic machinery. Genome Res. 2015;25:1473–81.*

*8. McKusick-Nathans Institute of Genetic Medicine, Johns Hopkins. Online Mendelian Inheritance in Man, OMIM® [Internet]. 2020. Available from: https://omim.org/*

*9. Thijssen PE, Ito Y, Grillo G, Wang J, Velasco G, Nitta H, et al. Mutations in CDCA7 and HELLS cause immunodeficiency–centromeric instability–facial anomalies syndrome. Nat Commun. 2015;6:7870.*

*10. Zhu H, Wang G, Qian J. Transcription factors as readers and effectors of DNA methylation. Nat Rev Genet. 2016;17:551–65.*

*11. Kawabe Y, Seki M, Seki T, Wang W-S, Imamura O, Furuichi Y, et al. Covalent Modification of the Werner’s Syndrome Gene Product with the Ubiquitin-related Protein, SUMO-1. J Biol Chem. 2000;275:20963–6.*

*12. Yannone SM, Roy S, Chan DW, Murphy MB, Huang S, Campisi J, et al. Werner Syndrome Protein Is Regulated and Phosphorylated by DNA-dependent Protein Kinase. J Biol Chem. 2001;276:8.*

*13. Kobayashi KS, van den Elsen PJ. NLRC5: a key regulator of MHC class I-dependent immune responses. Nat Rev Immunol. 2012;12:813–20.*

*14. Tornatore L, Thotakura AK, Bennett J, Moretti M, Franzoso G. The nuclear factor kappa B signaling pathway: integrating metabolism with inflammation. Trends Cell Biol. 2012;22:557–66.*

*15. Brion M-JA, Shakhbazov K, Visscher PM. Calculating statistical power in Mendelian randomization studies. Int J Epidemiol. 2013;42:1497–501.*

*16. Cedar H, Bergman Y. Linking DNA methylation and histone modification: patterns and paradigms. Nat Rev Genet. 2009;10:295–304.*

*17. Bonizzi G, Karin M. The two NF-κB activation pathways and their role in innate and adaptive immunity. Trends Immunol. 2004;25:280–8.*

*18. Luijk R, Dekkers KF, van Iterson M, Arindrarto W, Claringbould A, Hop P, et al. Genome-wide identification of directed gene networks using large-scale population genomics data. Nat Commun. 2018;9:3097.*

*19. Lemire M, Zaidi SHE, Ban M, Ge B, Aïssi D, Germain M, et al. Long-range epigenetic regulation is conferred by genetic variation located at thousands of independent loci. Nat Commun. 2015;6:6326.*

*20. Huan T, Joehanes R, Song C, Peng F, Guo Y, Mendelson M, et al. Genome-wide identification of DNA methylation QTLs in whole blood highlights pathways for cardiovascular disease. Nat Commun. 2019;10:4267.*

*21. Bonder MJ, Luijk R, Zhernakova DV, Moed M, Deelen P, Vermaat M, et al. Disease variants alter transcription factor levels and methylation of their binding sites. Nat Genet. 2017;49:131–8.*

*22. Gamazon ER, Wheeler HE, Shah KP, Mozaffari SV, Aquino-Michaels K, Carroll RJ, et al. A gene-based association method for mapping traits using reference transcriptome data. Nat Genet. 2015;47:1091–8.*

*23. Gusev A, Ko A, Shi H, Bhatia G, Chung W, Penninx BWJH, et al. Integrative approaches for large-scale transcriptome-wide association studies. Nat Genet. 2016;48:245–52.*

*24. Lawlor DA, Harbord RM, Sterne JAC, Timpson N, Davey Smith G. Mendelian randomization: Using genes as instruments for making causal inferences in epidemiology. Stat Med. 2008;27:1133–63.*

*25. The PRACTICAL consortium, Mancuso N, Gayther S, Gusev A, Zheng W, Penney KL, et al. Large-scale transcriptome-wide association study identifies new prostate cancer risk regions. Nat Commun. 2018;9:4079.*

*26. Davey Smith G, Hemani G. Mendelian randomization: genetic anchors for causal inference in epidemiological studies. Hum Mol Genet. 2014;23:R89–98.*

*27. Tibshirani R. Regression Shrinkage and Selection via the Lasso. J R Stat Soc Ser B. 1996;58:267–88.*

*28. van Iterson M, van Zwet EW, Heijmans BT. Controlling bias and inflation in epigenome- and transcriptome-wide association studies using the empirical null distribution. Genome Biol. 2017;18:19.*

*29. Wang K, Huang J. A Score-Statistic Approach for the Mapping of Quantitative-Trait Loci with Sibships of Arbitrary Size. Am J Hum Genet. 2002;13.*

*30. Pain O, Dudbridge F, Ronald A. Are your covariates under control? How normalization can re-introduce covariate effects. Eur J Hum Genet. 2018;26:1194–201.*

*31. Peng B, Yu RK, DeHoff KL, Amos CI. Normalizing a large number of quantitative traits using empirical normal quantile transformation. BMC Proc. 2007;1:S156.*

**Second round of review**

**Reviewer 1**

The authors have addressed my queries satisfactorily. I can recommend publication of this interesting work.

**Reviewer 2**

The authors addressed all issues and significantly improved the manuscript.
